# Contribution of Intrinsic Reactivity of the HIV-1 Envelope Glycoproteins to CD4-Independent Infection and Global Inhibitor Sensitivity

**DOI:** 10.1371/journal.ppat.1002101

**Published:** 2011-06-23

**Authors:** Hillel Haim, Bettina Strack, Aemro Kassa, Navid Madani, Liping Wang, Joel R. Courter, Amy Princiotto, Kathleen McGee, Beatriz Pacheco, Michael S. Seaman, Amos B. Smith, Joseph Sodroski

**Affiliations:** 1 Department of Cancer Immunology and AIDS, Dana–Farber Cancer Institute, Division of AIDS, Harvard Medical School, Boston, Massachusetts, United States of America; 2 Department of Chemistry, University of Pennsylvania, Philadelphia, Pennsylvania, United States of America; 3 Division of Viral Pathogenesis, Beth Israel Deaconess Medical Center, Boston, Massachusetts, United States of America; 4 Department of Immunology and Infectious Diseases, Harvard School of Public Health, Boston, Massachusetts, Unites States of America; University of Zurich, Switzerland

## Abstract

Human immunodeficiency virus (HIV-1) enters cells following sequential activation of the high-potential-energy viral envelope glycoprotein trimer by target cell CD4 and coreceptor. HIV-1 variants differ in their requirements for CD4; viruses that can infect coreceptor-expressing cells that lack CD4 have been generated in the laboratory. These CD4-independent HIV-1 variants are sensitive to neutralization by multiple antibodies that recognize different envelope glycoprotein epitopes. The mechanisms underlying CD4 independence, global sensitivity to neutralization and the association between them are still unclear. By studying HIV-1 variants that differ in requirements for CD4, we investigated the contribution of CD4 binding to virus entry. CD4 engagement exposes the coreceptor-binding site and increases the “intrinsic reactivity” of the envelope glycoproteins; intrinsic reactivity describes the propensity of the envelope glycoproteins to negotiate transitions to lower-energy states upon stimulation. Coreceptor-binding site exposure and increased intrinsic reactivity promote formation/exposure of the HR1 coiled coil on the gp41 transmembrane glycoprotein and allow virus entry upon coreceptor binding. Intrinsic reactivity also dictates the global sensitivity of HIV-1 to perturbations such as exposure to cold and the binding of antibodies and small molecules. Accordingly, CD4 independence of HIV-1 was accompanied by increased susceptibility to inactivation by these factors. We investigated the role of intrinsic reactivity in determining the sensitivity of primary HIV-1 isolates to inhibition. Relative to the more common neutralization-resistant (“Tier 2-like”) viruses, globally sensitive (“Tier 1”) viruses exhibited increased intrinsic reactivity, i.e., were inactivated more efficiently by cold exposure or by a given level of antibody binding to the envelope glycoprotein trimer. Virus sensitivity to neutralization was dictated both by the efficiency of inhibitor/antibody binding to the envelope glycoprotein trimer and by envelope glycoprotein reactivity to the inhibitor/antibody binding event. Quantitative differences in intrinsic reactivity contribute to HIV-1 strain variability in global susceptibility to neutralization and explain the long-observed relationship between increased inhibitor sensitivity and decreased entry requirements for target cell CD4.

## Author Summary

Human immunodeficiency virus (HIV-1), the cause of acquired immunodeficiency syndrome (AIDS), enters cells by attaching its major surface component, the envelope glycoprotein spike, to a receptor protein on the target cell called CD4. This attachment promotes cell entry by allowing the envelope protein to bind a second receptor (the “coreceptor”) on the cell. In rare cases, the virus is pre-activated and can bind coreceptor and enter cells in the absence of CD4. Such viruses, which are rarely found in patients, are also very sensitive to inhibition by antibodies made by the host immune system. We describe here a new property of HIV-1 called “intrinsic reactivity” that regulates the level of both entry into cells and sensitivity to antibodies. Intrinsic reactivity dictates the level of responsiveness of the envelope protein to the binding of stimulatory (coreceptor) and inhibitory (antibody) agents. Increases in intrinsic reactivity also result in enhanced sensitivity to inactivation at cold temperatures, which serves as a quantitative surrogate for intrinsic reactivity. In human hosts, HIV-1 must regulate its intrinsic reactivity to balance the ability to enter cells expressing low levels of CD4 with the requirement to avoid the host antibody response.

## Introduction

The entry of human immunodeficiency virus type 1 (HIV-1) into cells is mediated by the envelope glycoprotein complex on the viral membrane [1]. This complex is a trimer of heterodimeric subunits, each composed of a gp120 surface glycoprotein and a gp41 transmembrane glycoprotein [2]. In their unliganded form, the HIV-1 envelope glycoproteins exist in a high-potential-energy state. Binding to the receptors on the target cell triggers conformational changes in the envelope glycoproteins that lead to lower-energy states and activate the entry pathway [3], [4], [5].

Entry of most HIV-1 strains into cells is initiated by interaction of gp120 with the primary receptor, CD4, on the cell surface [6], [7]. The CD4-gp120 interaction induces significant conformational changes in the HIV-1 envelope glycoproteins [2], [3]. CD4 binding increases the ability of gp120 to engage the coreceptor, either CCR5 or CXCR4 [8], [9]. CD4 binding also induces the pre-hairpin intermediate, an envelope glycoprotein structure containing a trimeric coiled coil formed by the gp41 heptad repeat 1 (HR1) regions [10], [11], [12]. Subsequent binding of coreceptor to this CD4-activated intermediate leads to the formation of an energetically stable six-helix bundle, in which three gp41 heptad repeat 2 (HR2) helices interact with the HR1 coiled coil. During the process of receptor binding, the hydrophobic “fusion peptide” at the gp41 N-terminus is thought to penetrate into the target cell membrane [13], [14], [15]. As a result, the gp41 N-terminus and the transmembrane region are anchored in the target cell and viral membranes, respectively, and formation of the six-helix bundle thus approximates and fuses these membranes [11], [16], [17].

For most HIV-1 strains, CD4 is an obligate receptor. However, some naturally-occurring HIV-1 strains can replicate in cell types, like tissue macrophages or brain microglia, that express only low levels of CD4 [18]. Moreover, CD4-independent HIV-1 strains have been derived by virus passage on CD4-negative, coreceptor-positive cells in tissue culture [19], [20], [21], [22]. Despite the potentially advantageous broadening of tropism associated with a lower dependence on CD4 for infection, HIV-1 strains that are fully independent of CD4 have been identified only rarely in infected people [23], [24]. By contrast, CD4-independent strains of HIV-2 and simian immunodeficiency virus (SIV) are more commonly encountered in vivo, particularly in body compartments like the brain where cells expressing high levels of CD4 are limited in number [25], [26], [27], [28].

Studies of primate immunodeficiency virus variants have provided clues to the mechanism of CD4 independence and the balancing selection that operates on these viruses in vivo. Even in the absence of CD4, CD4-independent envelope glycoproteins can bind the coreceptor or antibodies that recognize “CD4-induced” gp120 epitopes overlapping the coreceptor-binding site (CoR-BS) [19], [20]. As expected, some of the sequence changes associated with acquisition of CD4 independence involve the CoR-BS itself (the V3 loop and β19 strand of gp120) or increase its exposure or formation (for example, by repositioning the V1/V2 loops that mask the coreceptor-binding region) [21], [29]. However, changes associated with CD4 independence have also been observed in envelope glycoprotein elements, including gp41, that are not directly involved in coreceptor binding [20], [22], [30]. How these changes influence viral dependence on CD4 is poorly understood.

Interestingly, most HIV-1, HIV-2 and SIV variants that are CD4-independent also exhibit an increased sensitivity to neutralization by multiple antibodies directed against different envelope glycoprotein epitopes [30], [31], [32], [33], [34]. Increased exposure of the CoR-BS may explain the increased sensitivity to antibodies that recognize CD4-induced or V3 gp120 epitopes, which overlap the CoR-BS [20], [22], [31], [32]. However, CD4-independent viruses also exhibit sensitivity to neutralization by antibodies that recognize epitopes not associated with coreceptor binding, such as the anti-gp41 antibody 2F5 and the 2G12 antibody, which recognizes gp120 glycans [30], [31]. For the latter epitopes, antibody binding to the envelope glycoprotein trimer has not demonstrated a consistent correlation with neutralization sensitivity or CD4 dependence [31], [33].

Generalized (global) changes in sensitivity to antibodies are not limited to CD4-independent viruses. In vitro adaptation of HIV-1 to replicate in tissue-cultured cells, in the absence of immune selective pressure, is often associated with an increase in sensitivity to multiple antibodies that target a wide range of envelope glycoprotein epitopes [35], [36], [37], [38], [39], [40], [41], [42]. In several instances, a limited number of sequence changes are associated with the generalized increase in sensitivity [32], [39], [40]. As observed for the neutralization-sensitive CD4-independent viruses, altered epitope integrity or exposure on the envelope glycoprotein trimer is not the sole explanation of the different sensitivity of HIV-1 variants to antibody neutralization.

To understand more clearly the role of CD4 in HIV-1 infection, we investigated the contribution of envelope glycoprotein changes to the CD4 independence and neutralization sensitivity of an HIV-1 variant selected to replicate in CD4^−^CCR5^+^ cells [19]. Several changes in the ectodomain of the gp41 transmembrane envelope glycoprotein were found to contribute to CD4 independence, even though they exerted little effect on CoR-BS exposure. These gp41 changes also contributed to the sensitivity of the envelope glycoprotein complex to a variety of perturbations (binding of antibodies and small molecules, exposure to cold). Thus, we propose that these gp41 changes modulate a previously unrecognized property of the HIV-1 envelope glycoproteins that we designate “intrinsic reactivity”. Intrinsic reactivity describes the propensity of the envelope glycoproteins to negotiate the transition to another state upon stimulation by ligand binding or incubation on ice. Our findings indicate that intrinsic reactivity and CoR-BS exposure both contribute to the formation/ exposure of the gp41 HR1 coiled coil, a structural change that is tightly correlated with CD4 independence. Furthermore, our results from a large panel of primary HIV-1 isolates suggest that intrinsic reactivity modulates the level of virus susceptibility to antibody-mediated neutralization. Thus, both coreceptor responsiveness and inhibitor sensitivity of HIV-1 are determined not only by the binding affinity of the relevant ligand, but by the reactivity of the envelope glycoprotein complex to the bound ligand.

## Results

### Determinants of CD4 Independence in the ADA/HXBc2 Chimeric Envelope Glycoproteins

The transition from CD4-dependent to CD4-independent HIV-1 typically involves multiple changes in gp120 and/or gp41 [21], [22], [29], [30]. However, in one case, the loss of a single glycosylation site due to an N197S change in the V1/V2 stem of gp120 was sufficient to generate a CD4-independent, neutralization-sensitive HIV-1 ADA variant [19]. This change increased the binding of unliganded gp120 both to CCR5 and to antibodies that recognize CD4-induced epitopes overlapping the CoR-BS [19]. The absence of a glycan at asparagine 197 was suggested to alter the conformation of the V1/V2 loops, which mask the CoR-BS [43]. In support of this model, deletion of the gp120 V1/V2 loops in either HIV-1 or SIV resulted in a CD4-independent phenotype [43], [44].

**Figure 1:**
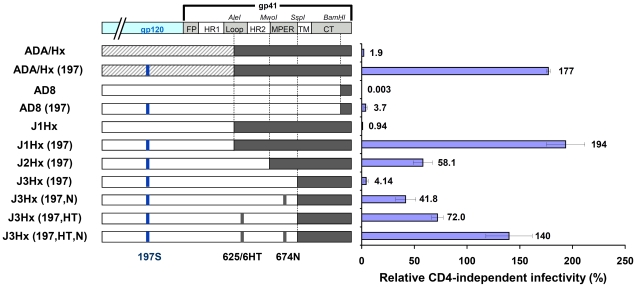
Identification of determinants of CD4 independence. Schematic representation of the chimeric envelope glycoprotein variants used in this study. Sequences derived from the ADA, HXBc2 and AD8 strains are represented by striped, black and white boxes, respectively. The infection of CD4^−^CCR5^+^ and CD4^+^CCR5^+^cells by 25,000 RT units of virus containing each envelope glycoprotein variant was measured. The relative CD4-independent infectivity of each envelope glycoprotein (± standard error of the mean, SEM) is defined as the level of infection of CD4^−^CCR5**^+^** cells divided by that of CD4**^+^**CCR5**^+^** cells.

Introduction of the N197S change into different HIV-1 strains did not result in CD4 independence [19]. We hypothesized that particular sequence elements in the envelope glycoprotein of the parental virus allowed the N197S mutant to replicate in CD4^−^CCR5^+^ cells. The ADA/Hx envelope glycoproteins used in the original study [19] are chimeric, with gp120 and the N-terminal part of the gp41 ectodomain derived from the CCR5-tropic ADA strain and the C-terminal part of gp41 derived from the CXCR4-tropic HXBc2 strain (Figure 1). To investigate the potential contribution of the HXBc2-derived gp41 sequences to CD4 independence, HIV-1 envelope glycoproteins that have the chimeric ADA/Hx gp41 ectodomain, the N197S change in gp120, or both modifications were tested for the ability to support virus entry into CD4^+^CCR5^+^ and CD4^−^CCR5^+^ cells. We compared the original ADA/Hx [19] with a new set of constructs made from the AD8 strain, which was molecularly cloned from the AD-87 derivative of ADA and closely resembles ADA in sequence (See Materials and Methods for details). Both the N197S change and the HXBc2-derived gp41 sequences (labeled J1Hx) increased the relative CD4-independent infectivity of the virus to a similar extent (Figure 1 and Figure S1A). Introduction of both changes exerted an even greater effect on infection of CD4^−^CCR5^+^ cells, allowing a level of infection comparable to that of the ADA/Hx envelope glycoproteins with the N197S change. Apparently, both the N197S and the gp41 changes in the ADA/Hx 197 envelope glycoproteins contribute to CD4 independence.

**Figure 2:**
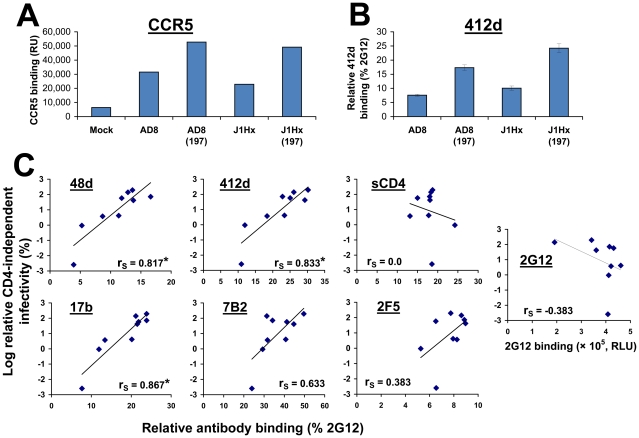
Epitope exposure on CD4-independent envelope glycoproteins. (**A**) Binding of radiolabeled wild-type and mutant gp120 to CD4^−^CCR5**^+^** Cf2Th cells. Following incubation of equivalent amounts of radiolabeled gp120 from the indicated HIV-1 envelope glycoprotein variants with CD4^−^CCR5^+^ cells at 37°C, the cells were washed and lysed. The lysates were used for immunoprecipitation of gp120, which was quantitated by densitometry. Data are presented as relative radioactivity units (RU). (**B**) Binding of the 412d antibody (1 µg/ml) to COS-1 cells that transiently express the indicated envelope glycoproteins at 37°C. The binding of 412d was normalized to that of a control anti-gp120 antibody, 2G12. (**C**) Relationship between the binding of antibodies and sCD4 to the cell surface-expressed envelope glycoproteins and the relative CD4-independent infectivity of each variant. The Spearman rank-order correlation coefficient is indicated (*, P<0.05).

We then examined the capacity of soluble gp120 shed from envelope glycoprotein-expressing cells to bind human CCR5 in the absence of CD4 (Figure 2A). As shown previously [19], [43], the N197S change increased CCR5 binding. The presence of the HXBc2-derived gp41 sequences did not affect the binding of shed gp120 to CCR5.

Exposure of the CoR-BS on the cell surface-expressed envelope glycoprotein trimer was measured using the 412d antibody, which mimics the N-terminus of CCR5 and recognizes a CD4-induced gp120 epitope [45], [46]. Binding was measured using a cell-based ELISA system [47] and normalized for envelope glycoprotein cell surface expression by comparison with the binding of the 2G12 antibody, which recognizes a gp120 carbohydrate epitope that is not involved in receptor engagement [48]. The binding of 412d to the envelope glycoprotein trimer was significantly increased by the N197S change, but was only slightly affected by the J1Hx gp41 changes (Figure 2B). These data indicate that the N197S change is sufficient for spontaneous exposure of the CCR5-binding site; however, both the N197S change and the HXBc2-derived gp41 sequences contribute to CD4-independent infection. Apparently, an envelope glycoprotein property that is distinct from CCR5 binding and contributes to infection of CD4^−^CCR5^+^ cells is specified by the gp41 sequences.

### Identification of the HXBc2-derived gp41 Sequence Elements that Contribute to CD4 Independence

To identify the HXBc2-derived sequence elements that impart CD4 independence to the J1Hx(197) chimera, we studied chimeric envelope glycoproteins that contain different portions of the HXBc2 gp41 ectodomain (See J1Hx(197), J2Hx(197), and J3Hx(197) in Figure 1). Reduction of the size of the gp41 ectodomain segment contributed by HXBc2 and replacement with the corresponding AD8 sequence resulted in a decreased capacity to infect CD4^−^CCR5^+^ cells (Figures 1 and S1A). The level of CD4-independent infection mediated by the J3Hx(197) construct was similar to that observed for the AD8(197) variant. These observations suggest that HXBc2 sequences in the gp41 ectodomain must contribute to the ability of the chimeric envelope glycoproteins to support CD4-independent infection.

Several AD8 amino acid residues in the implicated gp41 ectodomain segment were altered to the corresponding HXBc2 residues. We thus identified two changes (NM625/626HT and D674N (hereafter designated HT and N, respectively)) that significantly contribute to the capacity to infect CD4^−^CCR5^+^ cells (Figure 1 and Figure S1A). In the context of the J3Hx(197) envelope glycoproteins, the HT and N changes additively contributed to CD4-independent infection (Figure 1 and Figure S1A), together explaining most of the contribution of the HXBc2 ectodomain sequences to this phenotype. Similarly, the HT and N changes contributed to CD4-independent infection when introduced into the AD8(197) envelope glycoproteins (data not shown).

No effects of the N197S or gp41 changes on the efficiency of gp160 envelope glycoprotein precursor processing, cell-surface expression level or virion incorporation that correlated with CD4 independence were observed (Figures S1B and S1C). However, the CD4 independence of the envelope glycoprotein variants strongly correlated with the binding of the 17b, 48d and 412d antibodies, which are directed against CD4-induced gp120 epitopes [49], to the unliganded cell surface-expressed envelope glycoproteins (Figure 2C). By contrast, recognition and neutralization by the PG16 antibody, which preferentially binds the unliganded envelope glycoprotein trimer [50], were decreased for the more CD4-independent envelope glycoproteins (Figure S2). These results suggest that CD4-independent envelope glycoprotein trimers spontaneously sample conformations distinct from the unliganded state and closer to the CD4-bound state.

We examined the effects of the above gp41 changes in combination with deletion of the V1/V2 loops. Removal of the V1/V2 loops increased infection of CD4^−^CCR5^+^ cells and decreased infection of CD4^+^CCR5^+^ cells (Figure S3), similar to the effect of the N197S change. The HXBc2 gp41 ectodomain sequences contributed to the efficiency with which the V1/V2-deleted envelope glycoproteins supported entry into CD4^−^CCR5^+^ cells (compare AD8(ΔV) and J1Hx(ΔV) in Figure S3). The HT and N changes in gp41 also increased the relative CD4 independence of the J3Hx(ΔV) construct, but to a lesser extent than that observed for the J3Hx(197) envelope glycoproteins.

In summary, an HIV-1 envelope glycoprotein function specified by a small number of amino acid residues in the HxBc2 gp41 ectodomain contributes, along with increased exposure of the CoR-BS in gp120, to infection of CD4^−^CCR5^+^ cells.

### Spontaneous Formation/Exposure of the HR1 Coiled Coil in CD4-independent Envelope Glycoproteins

Binding of CD4 to the HIV-1 envelope glycoproteins results in the formation/exposure of two functionally significant structures: i) the CoR-BS on gp120, and ii) the gp41 HR1 coiled coil [49], [51], [52], [53], [54], [55]. CD4-independent envelope glycoproteins exhibit increased exposure of the CoR-BS [20], [30], [31], [34], [43], [44], but the state of gp41 in these envelope glycoproteins is not understood. We examined the formation/exposure of the HR1 coiled coil in our panel of envelope glycoprotein variants by using the C34-Ig molecule, which is composed of an IgG1 Fc region linked to the C34 peptide [53]. The C34 peptide corresponds to the gp41 HR2 region, which interacts with the HR1 coiled coil to form the six-helix bundle [16], [17]. To facilitate detection of bound C34-Ig, cell-surface expression levels of the HIV-1 envelope glycoproteins were increased by deletion of the cytoplasmic tail of gp41, which contains an endocytosis signal [56], [57]. Removal of the cytoplasmic tail did not detectably affect the conformation of the CD4-dependent AD8 envelope glycoproteins, and slightly increased the formation/exposure of the already-exposed CD4-induced epitopes on the highly CD4-independent envelope glycoproteins (Table S1). Furthermore, cytoplasmic tail deletion did not alter the relative ability of the different HIV-1 envelope glycoprotein variants to infect CD4^−^CCR5^+^ cells (Figure S4A).

**Figure 3:**
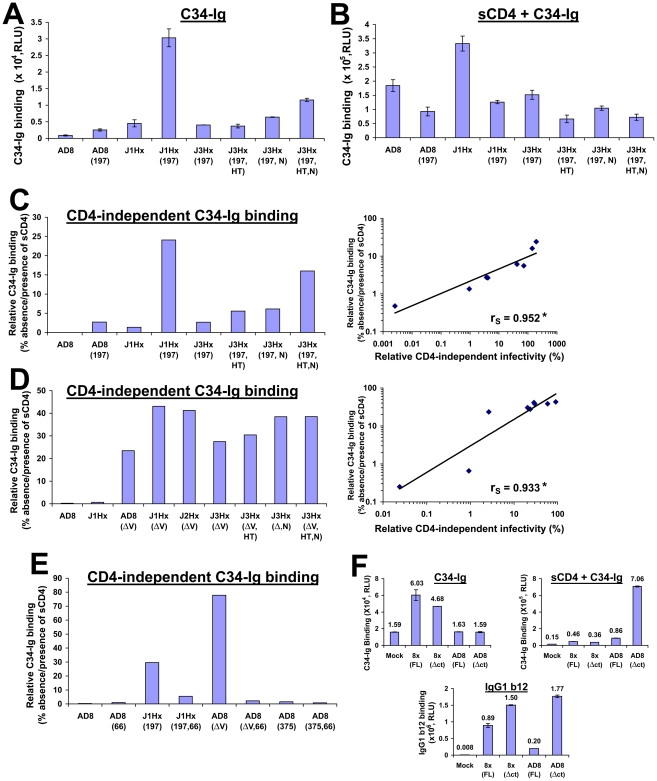
Relationship between spontaneous formation/exposure of the gp41 HR1 coiled coil and CD4 independence. (A,B) COS-1 cells that transiently express the indicated cytoplasmic tail-deleted envelope glycoproteins were incubated with 34 Ig(30 µg/ml) at 16°C for 30 min in the absence (A) or presence (B) of sCD4 (20 µg/ml). Data represent mean C34-Ig binding (± SEM) after subtracting the background binding of C34-Ig to mock-transfected cells. (C, left) Relative CD4-independent binding of C34-Ig, defined as the level of C34-Ig binding in the absence of sCD4 expressed as the percentage of C34-Ig binding in the presence of sCD4 for the panel of cytoplasmic tail- deleted envelope glycoprotein variants that combine the N197S mutation with the gp41 changes. (C, right) Correlation between relative CD4-independent infectivity mediated by the envelope glycoprotein variants and their relative CD4-independent binding of C34-Ig. The Spearman rank-order correlation coefficient is indicated (*, P<0.05). (D, left) Relative CD4-independent C34-Ig binding to cells expressing cytoplasmic tail-deleted envelope glycoprotein variants that combine a deletion of the V1/V2 loops with the gp41 changes. (D, right) Correlation between relative CD4-independent infectivity of V1/V2 loop-deleted envelope glycoproteins and their relative CD4-independent binding of C34-Ig. The Spearman rank-order correlation coefficient is indicated (*, P<0.05). (E) Effect of the H66N change (indicated by 66) and the S375W change (indicated by 375) on the relative CD4-independent binding of C34-Ig (30 µg/ml) to COS-1 cells expressing the indicated cytoplasmic tail-deleted envelope glycoprotein variants at 16°C. The deletion of the gp120 V1/V2 variable loops is indicated by ΔV. (F) C34-Ig binding to the envelope glycoproteins of the HIV-1 8x strain. COS-1 cells that transiently express the indicated envelope glycoproteins were incubated with C34-Ig (40 µg/ml) in the absence or presence of sCD4 (20 µg/ml) at 25°C. The cytoplasmic tail of the “full-length” (FL) 8x envelope glycoprotein is truncated and is composed of 27 amino acid residues (see ref. [29]). To eliminate any potential bias caused by cytoplasmic tail length, C34-Ig binding was measured to the full-length and truncated (Δct) form of both HIV-1 AD8 and 8x envelope glycoproteins. The cytoplasmic tail of both Δct envelope glycoproteins contains one amino acid (truncated after Asn 706). Binding of mAb IgG1 b12 (1 µg/ml) was also measured (bottom panel). Values represent means (± SEM) derived from duplicate samples.

Binding of C34-Ig to COS-1 cells that express the panel of mutant HIV-1 envelope glycoproteins was measured using the cell-based ELISA method [47]. Because binding of C34-Ig to the HIV-1 envelope glycoproteins is reduced at higher temperatures (Figure S4B), all binding assays were performed at temperatures below 26°C. Minimal C34-Ig binding to the unliganded AD8 envelope glycoproteins was detected, whereas treatment with sCD4 significantly increased C34-Ig binding, as expected (Figure 3A and B, and ref. [47]). Surprisingly, for the envelope glycoproteins with either the N197S change or the gp41 changes, C34-Ig binding could be detected even in the absence of sCD4, and was further increased by sCD4 treatment (Figure 3A–C). The envelope glycoprotein variants with both N197S and gp41 changes exhibited the highest levels of spontaneous C34-Ig binding in the absence of sCD4. The degree of C34-Ig binding to the envelope glycoprotein variants in the absence of sCD4 strongly correlated with the ability of the envelope glycoproteins to mediate infection of CD4^−^CCR5^+^ cells (Figure 3C, right panel). Thus, both the N197S change and the gp41 changes contribute to the formation/exposure of the HR1 coiled coil on gp41, which in turn promotes the infection of CD4-negative cells expressing CCR5.

We also studied C34-Ig binding to the panel of envelope glycoprotein variants with a deletion of the V1/V2 variable loops. V1/V2 loop deletion, even in the absence of any gp41 changes, induced an increase in C34-Ig binding far greater than that associated with the N197S or the gp41 changes (Figure 3D and Figure S4, C and D). C34-Ig binding to the unliganded V1/V2 loop-deleted envelope glycoproteins ranged from 20–45% of that seen after sCD4 treatment. Again, a good correlation was observed between C34-Ig binding to the unliganded envelope glycoproteins and the ability to mediate infection of CD4^−^CCR5^+^ cells (Figure 3D, right panel). This observation further supports the importance of formation/exposure of the gp41 HR1 coiled coil to the infection pathway for CD4^−^CCR5^+^ cells.

We also examined the effect of two previously characterized [58], [59] gp120 changes, S375W and H66N, on the binding of C34-Ig to the HIV-1 envelope glycoproteins. The S375W change increases the spontaneous sampling of the CD4-bound conformation by the HIV-1 gp120 glycoprotein, and modestly increases the formation/exposure of the CoR-BS [59]. The S375W change had no significant effect on the formation/exposure of the HR1 coiled coil (Figures 3E and S5B) and did not increase the ability of HIV-1 to infect CD4^−^ cells [see below and reference [59]. The H66N change has been shown to decrease the spontaneous exposure of the CoR-BS on the ADA/Hx(197) envelope glycoproteins [58]. The H66N change reduced the efficiency of CD4-independent infection mediated by both the ADA/Hx(197) and J1Hx(197) envelope glycoproteins ([58] and Figure S5A, respectively). The H66N change significantly reduced C34-Ig binding to the unliganded J1Hx(197) and AD8(ΔV) envelope glycoproteins (Figures 3E and S5B). These observations support the correlation between the ability of the HIV-1 envelope glycoproteins to mediate CD4-independent infection and spontaneous formation/exposure of the gp41 HR1 coiled coil.

Binding of sCD4 to the HIV-1 envelope glycoproteins induces a short-lived activated state, characterized by transient exposure of the HR1 coiled coil that coincides with a transient capacity to infect CD4^−^CCR5^+^ cells [47]. We examined how long after a sCD4 pulse the envelope glycoproteins remained competent for C34-Ig binding at 37°C. For the more CD4-independent envelope glycoproteins, the CD4-induced intermediates capable of binding C34-Ig exhibited longer half-lives (Figure S5C).

To learn whether the HR1 coiled coil is exposed on the unliganded envelope glycoproteins of other CD4-independent HIV-1 strains, we measured binding of C34-Ig to HIV-1 8x, a CXCR4-tropic, CD4-independent variant that was derived from the CD4-dependent HIV-1 IIIB strain [20]. As was observed for CD4-independent variants of the AD8 strain, the HIV-1 8x envelope glycoproteins bound C34-Ig in the absence of sCD4 (Figure 3F). By contrast, C34-Ig binding to the HXBc2 envelope glycoproteins, a CD4-dependent clone of the HIV-1 IIIB strain, required sCD4 addition (Figure S4D).

Thus, unliganded CD4-independent HIV-1 envelope glycoproteins are more prone to sample conformations that resemble the CD4-bound state, in which the HR1 coiled coil is formed and exposed. The spontaneous formation/exposure of this gp41 structure strongly correlates with the ability of the HIV-1 envelope glycoproteins to infect CD4-negative cells expressing CCR5.

### A Globally Inhibitor-sensitive State Is Induced by Specific Envelope Glycoprotein Changes Associated with CD4 Independence

Relative to the parental strains, many CD4-independent HIV-1 isolates exhibit an increased sensitivity to neutralization by antibodies [30], [31], [32]. The defined gradation of CD4 independence in our panel of AD8 variants provided an opportunity to examine the potential relationships between CD4 independence and sensitivity to neutralization by different antibodies and inhibitory ligands.

**Figure 4:**
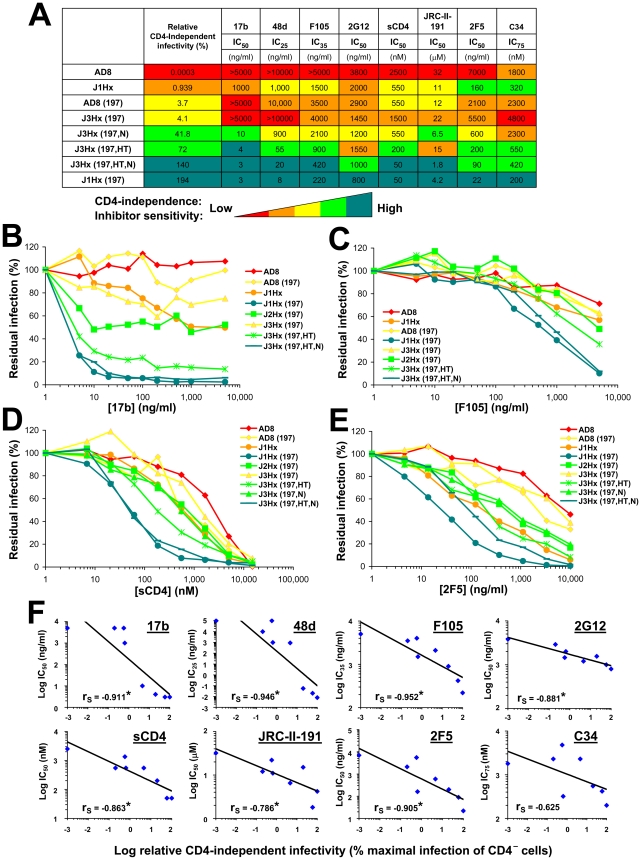
Association of CD4 independence and global neutralization sensitivity. (**A**) Sensitivity of viruses containing the indicated envelope glycoprotein variants **to** different inhibitors. The relative infectivity of each envelope glycoprotein variant for CD4^−^CCR5^+^ cells is shown for comparison. The relative levels of both CD4 independence and neutralization sensitivity are color coded as indicated. (**B–E**) Neutralization of viruses with the indicated envelope glycoproteins by sCD4 and antibodies. Each envelope glycoprotein variant is color coded according to its relative CD4-independent infectivity. (**F**) Correlation between the relative CD4-independent infectivity of envelope glycoprotein variants and sensitivity to neutralization. The Spearman rank-order correlation coefficient is indicated (*, P<0.05).

Regardless of the target epitope or inhibitor used, a similar general pattern of sensitivity to inhibition was observed (Figure 4, A–E and Figure S6). Viruses with the J1Hx(197) envelope glycoproteins were highly sensitive to multiple different inhibitory ligands compared with viruses containing the AD8 envelope glycoproteins. The N197S and gp41 changes cooperatively increased sensitivity to all inhibitors tested, regardless of the target epitope. The HT and N changes in gp41, either individually or together, positively affected this global neutralization sensitivity. The gp41 changes also exerted specific effects on neutralization by the gp41-directed 2F5 antibody; these local effects slightly perturbed the rank order of sensitivity of the envelope glycoprotein variants to this antibody. Despite these occasional epitope-specific effects, statistically significant correlations were observed between the level of CD4 independence of each envelope glycoprotein variant and sensitivity to inhibition by most of the ligands tested (Figure 4F). Of note, this correlation also applies to JRC-II-191, a 356-Dalton CD4-mimetic compound.

**Figure 5:**
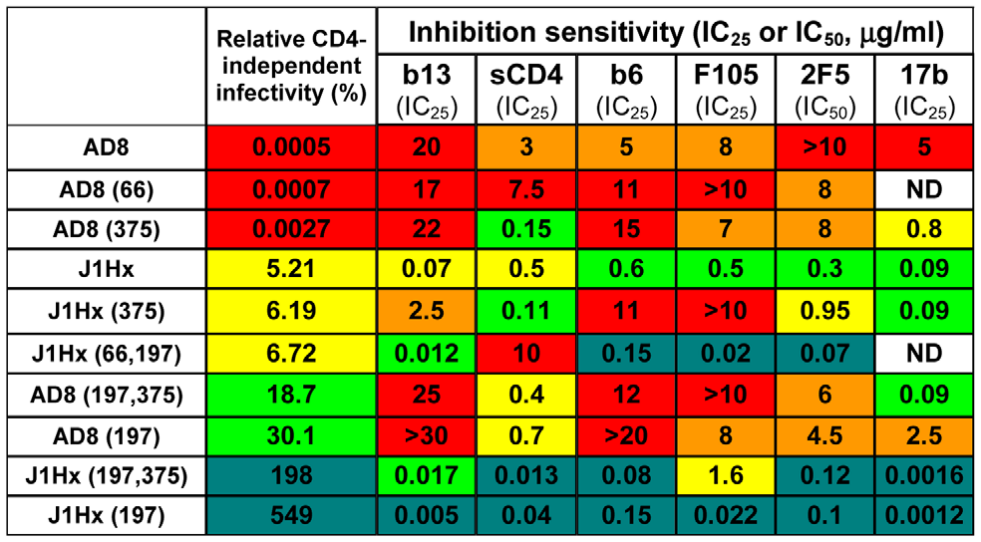
Effect of the H66N and S375W changes on neutralization sensitivity. Sensitivity of viruses containing the indicated envelope glycoprotein variants to the different inhibitors was measured and an IC_50_ or IC_25_ value determined. The capacity of each envelope glycoprotein variant to mediate infection of CD4^−^CCR5^+^ cells, relative to the ability to mediate infection of CD4^+^CCR5^+^ cells, is indicated for comparison. The relative levels of both CD4 independence and sensitivity to neutralization by each inhibitor are color-coded, as in Figure 4A. ND, not determined.

HIV-1 has evolved steric and thermodynamic constraints on the binding of antibodies to the CD4-binding site (CD4BS) of gp120 [60], [61]. Thus, most CD4BS antibodies, like F105, b6 and b13, bind envelope glycoprotein trimers inefficiently and neutralize HIV-1 only weakly. Based on x-ray structures of Fab complexes with monomeric gp120 cores, the target epitopes and approach angles of these weak CD4BS antibodies differ only subtly from those of the potent and broad CD4BS antibody, b12, which efficiently binds the envelope glycoprotein trimer [61]. The N197S change alone significantly decreased HIV-1 neutralization by the b12 antibody (Figure S6F); this result is consistent with previous observations that alterations in the gp120 V1/V2 loops can decrease HIV-1 sensitivity to b12 neutralization [36]. Because of the apparently local effects of the N197S change on envelope glycoprotein recognition by the b12 antibody, we compared the effect of the gp41 changes alone on virus neutralization by the above CD4BS antibodies. The sensitivity of HIV-1 to the b13, b6, F105 and b12 antibodies was enhanced by the gp41 changes (Figure 5 and Figure S6G). Of the different CD4BS antibodies, the b12 antibody neutralized the J1Hx virus, which has all of the gp41 changes, most potently. Thus, the differential potency of CD4BS antibodies, which is dictated by the efficiency with which envelope glycoprotein trimer binding occurs [61], applies in the context of globally sensitive envelope glycoproteins. In summary, the neutralization of this panel of viruses depends upon both the efficiency of antibody binding to the envelope glycoprotein trimer and a function dictated by the gp41 changes that results in global virus sensitivity to the bound antibody.

A similar correlation between CD4 independence and sensitivity to neutralization was observed for the panel of envelope glycoprotein variants with the V1/V2 variable loops deleted (Figure S7). Sensitivity of the viruses to both sCD4 and the 2F5 antibody was cooperatively determined by the gp41 changes and the deletion of the V1/V2 loops. As expected [62], all of the envelope glycoprotein variants that contain a deletion of the V1/V2 loops exhibited greater sensitivity to 2G12 neutralization due to increased binding of this antibody.

We also examined the effect of the H66N and S375W gp120 changes that respectively decrease and increase the spontaneous sampling of the CD4-bound state by the HIV-1 envelope glycoproteins [58], [59], [63], [64]. As shown above (Figure S5A and B), the H66N change decreases the efficiency of CD4-independent infection; by contrast, the S375W change exerted little specific impact on CD4 independence (Figure S8A). In accordance with previous observations [58], the H66N change reduced HIV-1 sensitivity to sCD4-mediated inhibition (Figure S8B). However, the H66N change did not affect the sensitivity of HIV-1 to CD4BS antibodies or to the 2F5 antibody (Figure 5 and Figure S8, C–F). The S375W change increased viral sensitivity to inhibition by sCD4 and the 17b antibody, but did not significantly affect sensitivity to the b6, b12, F105 or 2F5 antibodies. Therefore, the H66N and S375W changes specifically affect HIV-1 sensitivity to ligands like sCD4 and 17b that preferentially recognize the CD4-bound state. However, in contrast to the gp120 and gp41 changes associated with CD4 independence, the H66N and S375W changes do not globally affect the sensitivity of HIV-1 to inhibition.

### Inhibitor Binding to the HIV-1 Envelope Glycoprotein Variants

**Figure 6:**
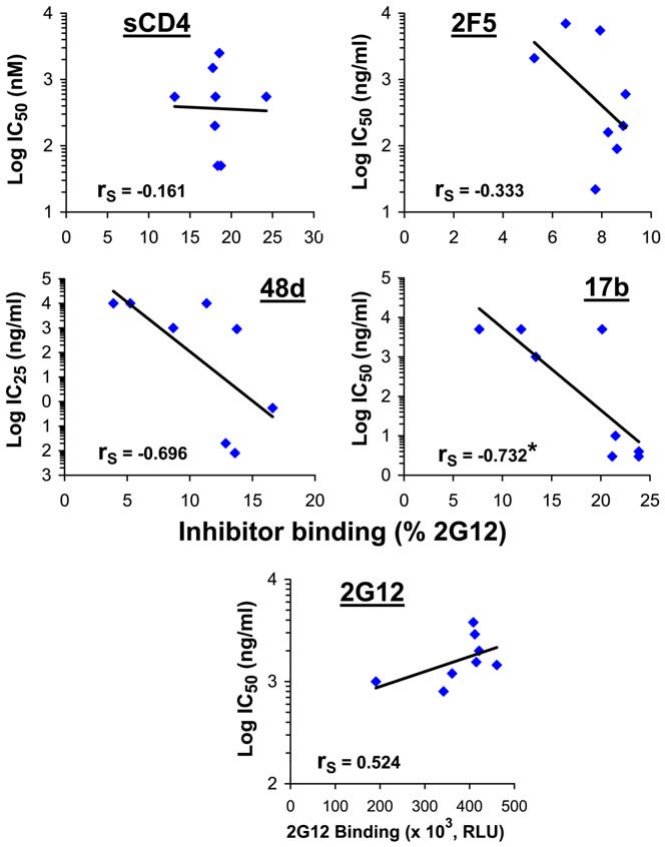
Relationship between neutralization sensitivity and inhibitor binding. The binding of inhibitors to the indicated envelope glycoproteins expressed on the cell surface was measured at 37°C. To normalize for cell-surface expression, inhibitor binding was calculated as percent binding of the control 2G12 antibody. Correlations between inhibitor binding to each envelope glycoprotein variant and the inhibitor IC_50_ or IC_25_ for viruses with the same envelope glycoprotein were examined. The Spearman rank-order correlation coefficient is indicated (*, P<0.05).

We examined whether the increased inhibitor sensitivity of viruses with CD4-independent envelope glycoproteins is a consequence of increased binding of the inhibitor to the envelope glycoprotein trimer. Despite significant differences among the envelope glycoprotein variant viruses in sensitivity to sCD4, 2F5 and 2G12, there was no evident correlation between inhibition sensitivity and steady-state binding of the inhibitor to the envelope glycoprotein trimer (Figure 6). Weak trends were observed for the CD4-induced antibodies 17b and 48d, which likely represent the effect of increased exposure of the CoR-BS induced by the N197S change. Accordingly, the N197S change increased the on-rate of 17b binding to the cell surface envelope glycoprotein trimer (Figure S9A).

We note that variation in inhibitor-induced shedding of gp120 from the envelope glycoprotein trimer did not explain differences in neutralization sensitivity of viruses with these envelope glycoprotein variants (Figure S9, B and C).

The above results indicate that the changes in the gp41 ectodomain, in particular the HT and N sequences, significantly increase HIV-1 sensitivity to neutralization by antibodies that recognize a variety of different envelope glycoprotein structures and by a CD4-mimetic compound. In most cases, the increased neutralization sensitivity cannot be explained by increases in antibody binding to the envelope glycoprotein trimer. These same gp41 changes are also responsible for the increased capacity of the envelope glycoproteins to infect cells in a CD4-independent manner (i.e., increased CCR5 responsiveness). In summary, both the gp41 changes and the deletion of the V1/V2 loops are responsible for the increased propensity of the HIV-1 envelope glycoproteins to negotiate spontaneously the transition into a conformation in which the HR1 coiled coil is formed and exposed. In this state the envelope glycoproteins have increased responsiveness to the CCR5 coreceptor. The gp41 changes and V1/V2 loop deletion also induce a state of increased reactivity to the binding of inhibitory ligands that target a wide range of envelope glycoprotein epitopes.

### Relationship between CD4 Independence and Cold Inactivation

**Figure 7:**
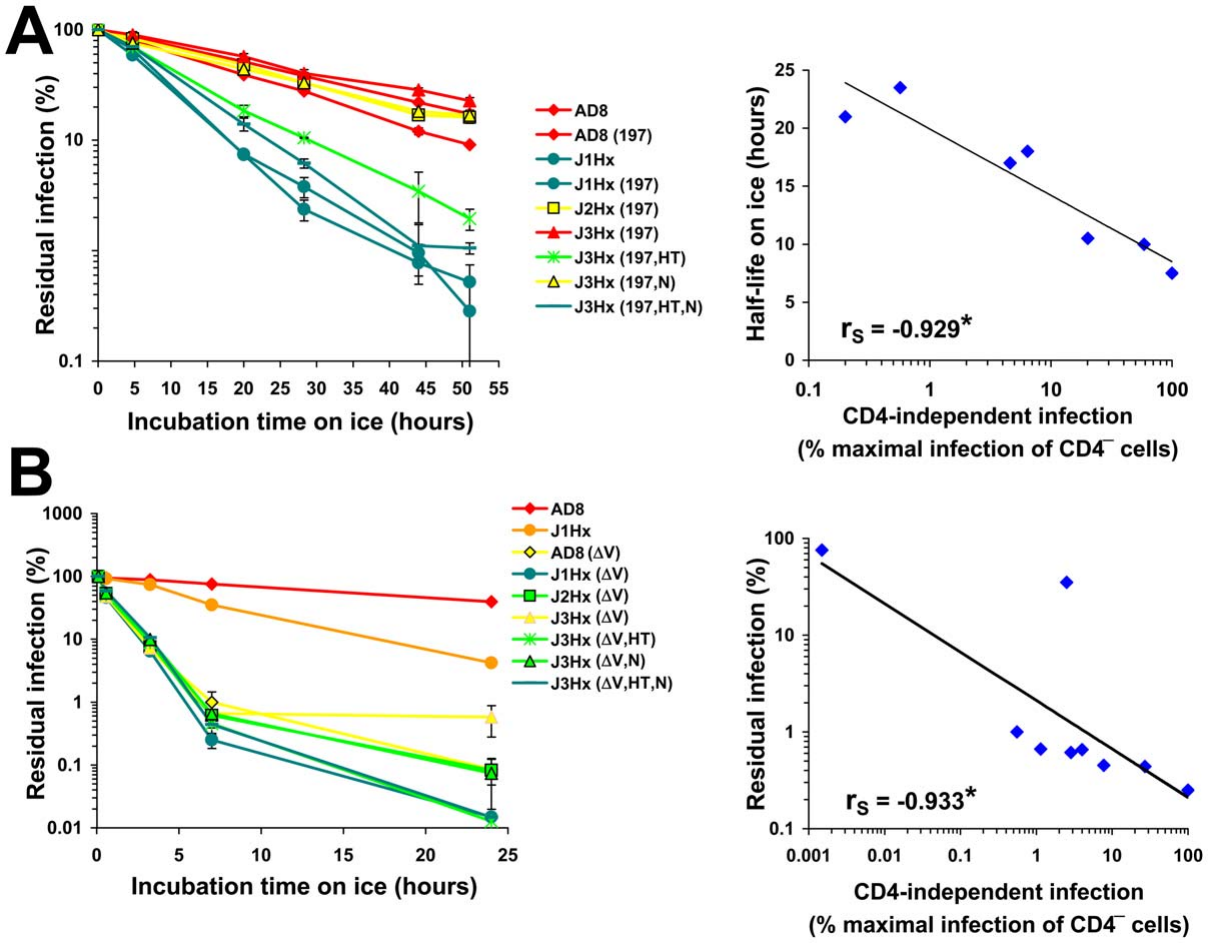
Relationship between CD4 Independence and cold sensitivity. (**A,B**) In the left panels, viruses containing the indicated envelope glycoproteins were incubated on ice for different times and then added to CD4^+^CCR5^+^ Cf2Th cells. The data represent mean (± SEM) levels of infection measured for each time point as a percent of the level of infection measured for viruses directly thawed from frozen stocks. Envelope glycoproteins are color coded according to relative CD4-independent infectivity. In the right panels, correlations are shown between CD4-independence (expressed as percent of the maximal infection of CD4^−^CCR5^+^ cells measured for the tested panel of variants) and cold resistance. For the correlations, cold resistance is expressed as half-life for the panel of envelope glycoprotein variants that contain the N197S change (**A**), or as the residual infectivity after a 7-hr incubation on ice (**B**). The Spearman rank-order correlation coefficient is indicated (*, P<0.05).

HIV-1 envelope glycoproteins that more frequently sample the CD4-bound state are more prone to inactivation by exposure to cold [63]. We therefore examined the possible linkage between CD4 independence and cold sensitivity. To measure cold sensitivity, we incubated virus on ice for different time periods and then added the virus to CD4^+^CCR5^+^ cells to measure infectivity. The J1Hx chimeric construct was highly sensitive to cold-induced inactivation, with or without the N197S change (Figure 7A, left panel). The main determinant of cold sensitivity was the gp41 HT change. The gp41 N change mildly increased sensitivity to cold (Figure S10A) and also enhanced the cold sensitivity of the HT variant. By contrast, the N197S change, which increases coreceptor-binding site exposure and enhances CD4-independent infection, exerted a slightly negative effect on cold sensitivity. For the group of N197S-containing envelope glycoprotein variants with different gp41 ectodomains, cold sensitivity correlated with the degree of CD4 independence (Figure 7A, right panel). This correlation is consistent with a model in which the same property that is dictated by the gp41 changes and that affects coreceptor reactivity and global inhibitor sensitivity is also responsible for increased sensitivity of the envelope glycoproteins to cold-induced inactivation.

Deletion of the V1/V2 loops resulted in a dramatic increase in cold sensitivity; the half-life on ice of approximately 19 hours for viruses with the AD8 envelope glycoproteins was reduced to 0.5 hours for the V1/V2-deleted mutant (Figure 7B, left panel). Combination of the V1/V2 deletion and the gp41 HT change further increased cold sensitivity. The cold sensitivity and CD4 independence of this panel of V1/V2-deleted envelope glycoproteins also exhibited a strong correlation (Figure 7B, right panel).

Changes in gp120 that were previously shown to decrease (H66N) or increase (S375W) gp120 sampling of the CD4-bound conformation [58], [59], [63] increased or decreased, respectively, the half-lives of the AD8 envelope glycoproteins on ice ([63] and Figure S10B). Similarly, incubation of the HIV-1 variants with sub-neutralizing concentrations of the CD4-mimetic compound JRC-II-191 resulted in increased cold sensitivity (Figure S10C). Removal of the cytoplasmic tail did not alter the pattern or absolute level of cold sensitivity of the envelope glycoprotein panel (Figure S10D).

Increased cold sensitivity was not associated with an increased propensity to shed gp120 from the envelope glycoprotein trimer. Incubation of viruses on ice did not result in release of gp120 from virions, as measured by the amount of residual infectivity in a virus capture assay (Figure S10E) or by changes in the level of gp120 in the supernatant (Figure S10F).

In summary, three alterations in the envelope glycoproteins (the HT and N changes in gp41 and the V1/V2 deletion in gp120) contributed to the capacity of HIV-1 to infect CD4^−^CCR5^+^ cells, but also increased virus susceptibility to neutralizing ligands and to cold-induced inactivation. We designate this property of increased sensitivity to coreceptor, inhibitors and cold temperatures “intrinsic reactivity”. Intrinsic reactivity describes the propensity of the HIV-1 envelope glycoproteins to negotiate transitions to another state upon perturbation by ligand binding or thermal stimulation. Of note, although HIV-1 envelope glycoprotein variants that exhibit increased intrinsic reactivity are more cold-sensitive, the converse is not true; some cold-sensitive envelope glycoproteins (e.g., S375W) are not globally sensitive to antibody neutralization and thus cannot be considered intrinsically reactive. Therefore, additional paths to the cold-sensitive state exist that do not involve an increase in intrinsic reactivity.

### Relationship between Inhibitor Binding and Inhibitor Efficiency in Envelope Glycoproteins Derived from Primary HIV-1 Isolates

in vivo propagation of HIV-1, in the absence of immune selective pressure, is often associated with a generalized increase in sensitivity to neutralization by antibodies [41], [65], [66]. Passage of simian-human immunodeficiency viruses containing the envelope glycoproteins of these laboratory-adapted HIV-1 strains in monkeys resulted in reversion of the neutralization-sensitive viruses to more resistant forms [67], [68]. Similarly, *in vitro* passage of HIV-1 in the presence of neutralizing antibodies also generated globally neutralization-resistant viruses [69], [70]. Global changes in sensitivity to antibodies are often induced by a limited number of changes in envelope glycoprotein sequence [32], [39], [40]. In several cases, the changes in neutralization sensitivity could not be explained solely on the basis of changes in binding affinity of the antibodies to the envelope glycoproteins [31], [33], [41], [70]. We hypothesized that, in such cases, alterations in the intrinsic reactivity of the envelope glycoproteins contribute to the observed changes in HIV-1 neutralization sensitivity.

The above-described chimeras between the envelope glycoproteins of AD8 and HXBc2 strains of HIV-1 were generated artificially. We sought to determine the potential role of intrinsic reactivity in the phenotypes of primary viruses cloned directly from HIV-1-infected individuals. For this purpose, we used a standardized panel of 16 envelope glycoproteins derived from diverse primary HIV-1 isolates, which serve as the reference strains for assessment of vaccine-elicited neutralizing antibodies [71]. These envelope glycoproteins have been classified on the basis of sensitivity of viruses containing them to neutralizing antisera: Tier 1 viruses are more sensitive than Tier 2 viruses to antibody neutralization [72]. In a recent study, the Tier 1 group of envelope glycoproteins was subdivided based on mean sensitivity of the associated viruses to six pools of human plasma into Tier 1A and Tier 1B classes [73].

To examine the basis for the increased neutralization sensitivity of Tier 1 HIV-1 isolates, we tested several inhibitors, including sCD4 and the monoclonal antibodies IgG1 b12, 2G12, and 4E10. A general trend of increased sensitivity of the Tier 1 viruses relative to the Tier 2 viruses was observed; 2-tailed T-test P values were 0.0495, 0.0884, 0.168 and 0.271 for sCD4, 2G12, 4E10 and IgG1 b12, respectively (Figure S11).

**Figure 8:**
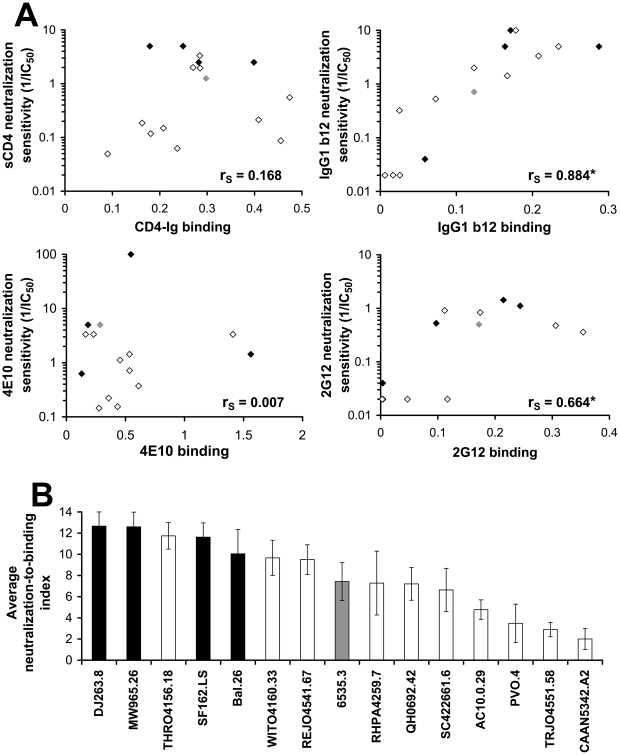
Relationship between inhibitor binding and inhibition efficiency in a standardized panel of primary HIV-1 isolates. (**A**) Binding of the indicated inhibitors to COS-1 cells expressing envelope glycoproteins from primary HIV-1 isolates was measured at 37°C using the cell-based ELISA; the antibodies IgG1 b12, 2G12 and 4E10 were added at a concentration of 1 µg/ml and CD4-Ig was added at 2 µg/ml. Binding was normalized for cell-surface expression measured by cell-surface immunoprecipitation from ^35^S-labeled cells (see Materials and Methods section). The relationship between neutralization sensitivity of viruses containing the envelope glycoproteins (given by the 1/IC50 value) and the normalized binding efficiency is shown. The envelope glycoproteins are colored according to neutralization sensitivity (Tier 1A, black; Tier 1B, grey; and Tier 2, empty symbols). The Spearman rank-order correlation coefficient is indicated (*, P<0.05). (**B**) A generalized measure of envelope glycoprotein sensitivity to the binding of inhibitors. For each envelope glycoprotein, the ratio of the neutralization sensitivity and binding efficiency of each of the inhibitors, as described in panel (**A**), was calculated (See Materials and Methods). For each inhibitor tested, the envelope glycoproteins were rank ordered according to their neutralization-to-binding ratios. Shown is the mean (± SEM) rank order of neutralization-to-binding for each of the envelope glycoproteins averaged for the four inhibitors. Color coding is the same as that in panel (**A**).

We asked whether the observed differences in susceptibility of the primary viruses to neutralization by these inhibitors results from the differential binding of the inhibitors to the envelope glycoproteins. For this purpose, we measured binding of the inhibitors to the envelope glycoprotein trimers using a cell-based ELISA. Binding data were normalized for the level of cell-surface expression of each envelope glycoprotein, which was determined by a cell-surface immunoprecipitation assay (see Materials and Methods). As shown in Figure 8A, significant correlations between binding to the envelope glycoprotein complexes and virus neutralization were observed for the monoclonal antibodies IgG1 b12 and 2G12. By contrast, for both the 4E10 antibody and sCD4, there was no clear correlation between inhibitor binding and neutralization potency.

The absence of a binding-inhibition correlation for some of the inhibitors suggested that an envelope glycoprotein property influencing susceptibility to the bound inhibitor might be contributing to the neutralization sensitivity of the virus. To examine this possibility, we calculated the ratio of neutralization sensitivity of the virus to the efficiency of ligand binding to the cell-surface envelope glycoproteins for each of the four inhibitors tested (Figure S12A–D). This neutralization-to-binding ratio quantitatively describes the “reactivity” of the envelope glycoproteins to the binding of inhibitors. The envelope glycoproteins of the panel were arranged in rank order of their neutralization-to-binding ratios, whereby the strain with the highest sensitivity was assigned the maximal value of 15 and the lowest a value of one. In addition, for each envelope glycoprotein, the mean of its relative rank order for the four inhibitors tested was calculated and is designated the average neutralization-to-binding index (Figure 8B). By averaging the rank orders rather than using absolute neutralization-to-binding ratios, we imparted an identical weight to the results obtained from each of the tested antibodies and thus eliminated potential bias introduced by any single value.

Viruses containing Tier 1 envelope glycoproteins had significantly higher average neutralization-to-binding indices than viruses containing Tier 2 envelope glycoproteins (Figure 8B). Two-tailed T-test P values were 0.007 for a comparison of Tier 1A with Tier 1B and Tier 2 envelope glycoproteins, and 0.018 for the comparison of Tier 1A and Tier 1B with Tier 2 envelope glycoproteins. The Tier 1B envelope glycoproteins from the 6535.3 HIV-1 strain, previously categorized as a Tier 2 virus [72], exhibited only a moderate level of sensitivity to inhibitor binding relative to the Tier 1A viruses. When a comparison between Tier 1 and Tier 2 viral envelope glycoproteins was made for the neutralization-to-binding ratios of the individual inhibitors rather than the averaged index, a statistically significant difference was found for sCD4 and a trend towards significance for the 4E10 antibody (Figure S12, A–D). These results suggest that envelope glycoproteins of primary HIV-1 strains differ in their reactivity to the binding of inhibitors; Tier 1 viruses are generally more sensitive than Tier 2 viruses to inhibitor binding. Thus, as shown for the panel of AD8 variants, not only the binding efficiency of the inhibitor but also the reactivity of the envelope glycoproteins to the binding event determines the level of HIV-1 susceptibility to the inhibitor.

We note that envelope glycoprotein cleavage status did not differ between the Tier 1 and Tier 2 isolates. We performed a cell-surface immunoprecipitation of the Tier 1/Tier 2 envelope glycoproteins using the b12, 2G12 and VRC01 antibodies. No significant differences in the gp120 to gp160 ratios for the cell surface fraction were observed between envelope glycoproteins of Tier 1 and Tier 2 isolates (data not shown). The efficiency with which the HIV-1 envelope glycoproteins are cleaved can influence the binding of the non-neutralizing CD4BS antibodies, b6 and b13, compared with that of the neutralizing CD4BS antibody b12 [61]. We therefore measured the binding of the b6, b13 and b12 antibodies to the primary envelope glycoproteins expressed on COS-1 cells using the cell-based ELISA. No differences were observed between the two Tiers in absolute levels of b6 or b13 binding or in their binding relative to the b12 antibody (data not shown). These results are in agreement with those obtained for the panel of AD8 variants that show no particular trend in the cleavage status of the intrinsically reactive envelope glycoproteins that would bias our results.

### Cold Sensitivity of Neutralization-Sensitive and -Resistant Primary HIV-1 Strains

**Figure 9:**
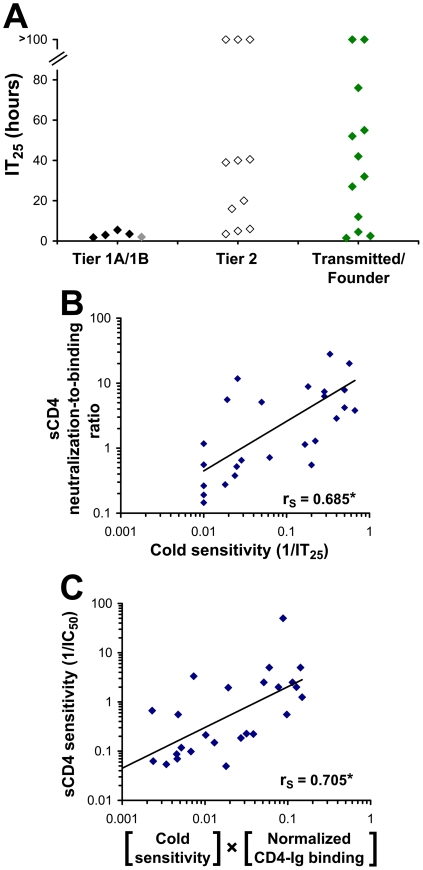
Relationship between cold sensitivity and inhibitor sensitivity of primary HIV-1 envelope glycoproteins. (**A**) Sensitivity to cold-induced inactivation of viruses containing envelope glycoproteins derived from a standardized panel of primary HIV-1 isolates, including transmitted/founder HIV-1. Data represent the mean incubation time on ice required for a 25% decrease from initial infectivity (IT25) in an experiment performed with three replicate samples. Color coding is the same as that in Figure 8. (**B**) Correlation between the cold sensitivity of viruses and their reactivity to the binding of sCD4. The neutralization-to-binding ratio is defined as the reciprocal of the IC50 value of the virus containing the envelope glycoproteins divided by the efficiency of sCD4 binding to the envelope glycoprotein trimer (normalized for the level of cell-surface expression). The 26 envelope glycoproteins included in the cohort are listed in the Materials and Methods section. *, Two-tailed T-Test P value 0.000112. (**C**) Correlation between sCD4 sensitivity and the product of CD4-Ig binding to cell-surface envelope glycoproteins and cold sensitivity. The sCD4 sensitivity of viruses with the envelope glycoproteins from the primary HIV-1 cohort was plotted versus the product of cold sensitivity and CD4-Ig binding to cell-surface envelope glycoproteins (normalized for cell-surface expression). *, Two-tailed T-Test value P value 0.00006.

We hypothesized that the increased sensitivity to inhibitor binding of the Tier 1 envelope glycoproteins is a consequence of an increased level of intrinsic reactivity. As our previous results indicated that intrinsically reactive envelope glycoproteins are cold-sensitive, we examined the cold sensitivity of the primary HIV-1 envelope glycoprotein panel. As shown in Figure 9A, all of the viruses pseudotyped with the Tier 1 envelope glycoproteins were highly sensitive to cold inactivation. By contrast, the Tier 2 viruses were more cold-resistant (two-sided P-value = 0.011, Wilcoxon-Mann-Whitney test). Tier 2 viruses exhibited significant variability in cold sensitivity; IT_25_ values ranged from 2 hours to more than 100 hours. We also examined the envelope glycoproteins derived from a panel of transmitted/founder HIV-1 isolates, which generally exhibit phenotypes characteristic of Tier 2 viruses [73]. Similar to the panel of Tier 2 envelope glycoproteins, the transmitted virus envelope glycoproteins also exhibited a wide range of sensitivities to cold inactivation.

Although Tier 1 envelope glycoproteins were highly cold-sensitive relative to the Tier 2 panel, there was only a weak trend between cold sensitivity and the level of global reactivity to inhibitor binding, as measured by the neutralization-to-binding index for the 4 inhibitors (Figure S13A). Therefore, although cold sensitivity can serve as a highly sensitive measure of a global neutralization-sensitive state, it is not specific for this state. This conclusion is consistent with the results obtained with the S375W and H66N changes in gp120, which affect cold sensitivity but not global neutralization sensitivity. Both gp120 changes, however, demonstrated specific effects on sensitivity to sCD4 (Figure 5). We therefore examined the panel of primary HIV-1 envelope glycoproteins for a relationship between sensitivity to cold and reactivity to bound sCD4 and monoclonal antibodies. Of the inhibiting ligands examined, only the sCD4 neutralization-to-binding ratio correlated with sensitivity to cold. This correlation was supported by examining a larger panel of envelope glycoproteins from 26 primary HIV-1 isolates, including Tier 1, Tier 2 and transmitted/founder isolates (see list in Materials and Methods) (Figure 9B). For this panel, sensitivity to sCD4 neutralization could be predicted by two factors: CD4 binding to the cell-surface envelope glycoproteins and cold sensitivity (Figure 9C). Interestingly, the relationship between the neutralization-to-binding ratio and cold sensitivity was more apparent for sCD4 and 4E10 than for IgG1 b12 and 2G12 (Figure S13B–E). This is consistent with envelope glycoprotein intrinsic reactivity playing a role in sCD4, and perhaps 4E10, neutralization. For such inhibitors, which are dependent upon both binding and intrinsic reactivity for inhibition, a lower level of correlation between binding and inhibition is expected. By contrast, for antibodies that are less dependent upon envelope glycoprotein reactivity for inhibition (such as IgG1 b12 and 2G12), binding efficiency to the envelope glycoprotein trimer is the dominant determinant and correlates well with neutralization sensitivity.

In summary, the neutralization-to-binding index serves as both a sensitive and specific indicator of the level of global sensitivity (reactivity) to inhibitors. By contrast, cold sensitivity serves as a sensitive measure of intrinsic envelope glycoprotein reactivity but has low specificity. The cold-sensitive state, regardless of the level of global neutralization sensitivity, is generally associated with an increased envelope glycoprotein reactivity to sCD4 binding.

## Discussion

Persistent viruses like the primate immunodeficiency viruses must avoid envelope glycoprotein-directed neutralizing antibodies. The use of two receptors for virus entry allows sequestration of the coreceptor-responsive (but also inhibitor-reactive) state from the humoral immune system of the host [74], [75]. The relatively neutralization-resistant unliganded viral envelope glycoprotein spike is activated by engagement of the primary receptor, CD4, and is thus primed for subsequent events on the entry pathway. At the virus-cell interface, the CD4-bound state is relatively stable and protected from most neutralizing antibodies by steric factors [75]; by contrast, the sCD4-bound state on free HIV-1 virions is labile and highly sensitive to antibody neutralization [33], [34], [47]. Viruses adapting to host compartments with different levels of target cell CD4 and neutralizing antibodies must balance envelope glycoprotein requirements for entry, antibody resistance and stability. Here, by studying a panel of HIV-1 envelope glycoproteins differing incrementally in CD4 dependence, we define the entry functions provided by CD4 binding, identify a novel property of the HIV-1 envelope glycoproteins and clarify how this property influences virus entry and sensitivity to inhibitors and environmental factors.

**Figure 10:**
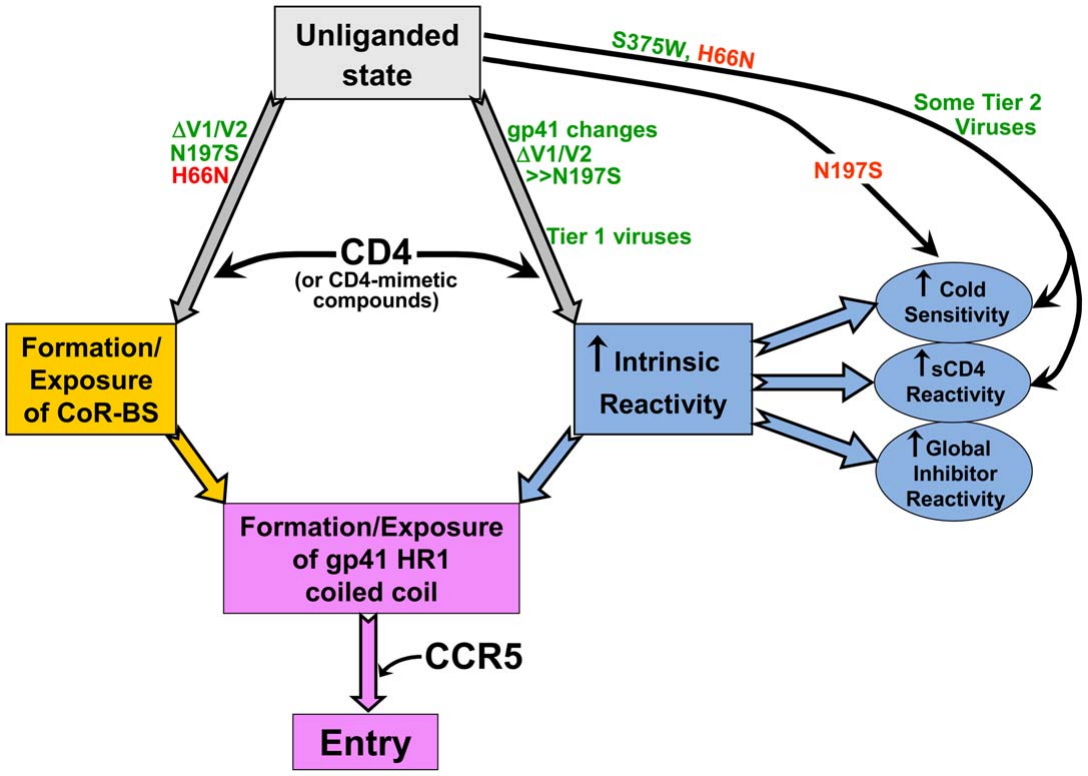
Early events in HIV-1 entry. The relationship among the early events in HIV-1 entry that typically are triggered by CD4 binding is depicted. Increased intrinsic reactivity and exposure of the CoR-BS cooperatively contribute to formation/exposure of the gp41 HR1 coiled coil. The points at which HIV-1 envelope glycoprotein changes act in either a positive (green) or negative (red) fashion are noted.

The phenotypic effects of the envelope glycoprotein changes and the correlations among these phenotypes suggest a model for the contributions of CD4 binding to HIV-1 entry (Figure 10). CD4 binding to gp120 facilitates two events, both of which contribute to the formation/exposure of the gp41 HR1 coiled coil and virus entry. One event is the formation and exposure of the gp120 binding site for CCR5 and/or CXCR4. CD4-independent envelope glycoproteins can spontaneously expose the CoR-BS, a state that is associated with increased recognition by antibodies directed against CD4-induced epitopes. A strong correlation exists between CD4 independence and exposure of these epitopes.

Changes in the gp41 ectodomain contributed to CD4 independence, global neutralization sensitivity, and cold susceptibility, yet exerted little or no effect on CoR-BS exposure or antibody binding to the envelope glycoprotein trimer. These observations suggest the existence of a property of the HIV-1 envelope glycoproteins that we designate “intrinsic reactivity.” Intrinsic reactivity describes the propensity of the envelope glycoproteins to undergo transitions from their native high-energy state to a lower-energy state upon stimulation. In the case of CD4-independent infection, enhanced intrinsic reactivity increases the probability that the unliganded HIV-1 envelope glycoproteins will make the transition to the pre-hairpin intermediate without the assistance of CD4. This is reflected in the positive effect of the gp41 changes on the spontaneous formation/exposure of the HR1 coiled coil. Enhanced intrinsic reactivity is also manifest as a global reactivity of HIV-1 to bound ligands (antibodies, sCD4 and small molecules) and to exposure to the cold; in these cases, the HIV-1 envelope glycoproteins apparently make transitions to non-functional states. Cold sensitivity indicates that intrinsic reactivity is an intrinsic property of the envelope glycoproteins and is not dependent on the presence of a ligand. The observed range of susceptibility of envelope glycoproteins to cold inactivation and the quantitative correlation of those values with CD4-independence indicate that intrinsic reactivity may assume multiple values (i.e., is not a binary variable).

Exposure of the CoR-BS and increases in intrinsic reactivity independently promoted the formation/exposure of the gp41 HR1 coiled coil. The ability of the HIV-1 envelope glycoprotein variants to bind the gp41 HR2 peptide (C34-Ig) in a spontaneous manner was tightly correlated with the ability to mediate CD4-independent infection. C34-Ig binding indicates that, in CD4-independent HIV-1 envelope glycoproteins, the HR1 coiled coil is either already formed or can readily be formed in the presence of soluble HR2 peptides. Exposure of the HR1 coiled coil presumably sets the stage for coreceptor binding to trigger subsequent events in the entry process, such as formation of the six-helix bundle.

Conformational transitions from the unliganded state of the HIV-1 envelope glycoprotein trimer presumably involve movement from a local energy well over an activation barrier and down a steep energy gradient [1], [3], [16], [17]. We suggest that increases in intrinsic reactivity may decrease this activation barrier by loosening the quaternary constraints that preserve the unliganded state. This perspective helps to explain the particular sensitivity of intrinsically reactive HIV-1 envelope glycoproteins to cold and to ligand binding. Cold denaturation of proteins often involves the formation of ice-like water structures surrounding hydrophobic interfaces in oligomeric protein complexes [76]. Any decrease in the quaternary associations that maintain the unliganded state may expose envelope glycoprotein elements that are otherwise buried in the trimer, rendering them available for interaction with water during cold inactivation. The binding of some ligands induces significant conformational changes in the HIV-1 envelope glycoproteins [3], [60] and may also predispose the HIV-1 envelope glycoproteins to move to lower-energy states.

Soluble CD4 binding induces major conformational changes in the envelope glycoprotein spike [2], driving the unliganded HIV-1 envelope glycoproteins into an unstable activated intermediate. The short-lived intermediate is characterized by increased exposure of the HR1 coiled coil and the capacity to infect CD4− cells [47]. Treatment with sCD4 or CD4-mimetic compounds also sensitizes the HIV-1 envelope glycoproteins to cold inactivation [58]. These observations hint that an increase in intrinsic reactivity may accompany CD4 binding and thus may represent a step in HIV-1 infection of CD4^+^ cells; from this perspective, an increase in intrinsic reactivity is not merely a means by which CD4-independent viruses bypass the requirement for CD4. Some V1/V2 loop-deleted envelope glycoprotein variants studied here exhibited spontaneous formation/exposure of the HR1 coiled coil that approached the level induced by sCD4 treatment; the functional viability of these variants suggests that the intermediate states sampled must exhibit at least modest stability. Of interest, the HXBc2 gp41 sequences associated with CD4 independence increased the longevity of HR1 coiled coil exposure following sCD4 treatment. These observations suggest that: 1) some CD4-independent viruses may have acquired changes that increase the stability of the envelope glycoproteins when assuming conformations that are near the CD4-bound state; and 2) the instability of the sCD4-activated envelope glycoproteins results from a process that is distinct from the formation/exposure of the HR1 coiled coil and is unnecessary for infection of CD4^−^CCR5^+^ cells. Therefore, although activation by CD4 during the entry process and inactivating events resulting from exposure to sCD4 or cold involve HIV-1 envelope glycoprotein transitions down an energy gradient, the resulting states differ in functionality. This separation explains how the sCD4-activated state of the SIV envelope glycoproteins can persist for several hours at 37°C [4], [5], [77] and how the envelope glycoproteins of some CD4-independent SIV strains exist constitutively in a conformation resembling the CD4-bound state [78].

The property of intrinsic reactivity explains the long-standing observation that in vivo or tissue-culture adaptation of HIV-1 to replicate in cells with low levels of CD4 results in increased sensitivity to neutralization by sCD4 and antibodies. At least two factors determine the outcome of the exposure of HIV-1 to an inhibitor directed against the envelope glycoproteins: a) the ability of the inhibitor to bind the functional envelope glycoprotein trimer; and b) the intrinsic reactivity of the viral envelope glycoproteins. Antibody binding to the envelope spike is a prerequisite for neutralization and, for HIV-1 variants with low intrinsic reactivity, represents the dominant determinant of neutralization potency. Epitope exposure contributes to the sensitivity of CD4-independent envelope glycoproteins to neutralization by the antibodies directed against CD4-induced gp120 epitopes. These were the only antibodies examined for which antibody binding to the envelope glycoprotein trimer exhibited any correlation with neutralization of the HIV-1 AD8 variants. For the other antibodies and inhibitors studied with this virus panel, altered intrinsic reactivity of the envelope glycoprotein is required to explain the observed differences in neutralization sensitivity. It is noteworthy that the CD4-BS antibody b12, which binds the HIV-1 envelope glycoprotein trimer more efficiently than the closely related b13 antibody [61], uniformly neutralized our panel of CD4-independent viruses better than b13. Thus, the envelope glycoprotein structures that influence the differential binding of these antibodies to the trimer [61] must be preserved over a range of intrinsic reactivities.

Our results suggest that intrinsic reactivity also influences the sensitivity of some primary HIV-1 strains to neutralization by antibodies or sCD4. By measuring the neutralization-to-binding ratios for individual antibodies and generating an averaged index, we found that the envelope glycoproteins of Tier 1 primary HIV-1 exhibit increased reactivity to the bound ligands, relative to Tier 2 viruses. The globally neutralization-sensitive Tier 1 envelope glycoproteins did not bind inhibitory ligands more efficiently than the globally-resistant (Tier 2-like) envelope glycoproteins. Negligible infection of CD4^−^CCR5^+^ cells was measured in the panel of primary virus isolates (data not shown). Furthermore, no significant differences in exposure of the coreceptor binding site was observed between Tier 1 and Tier 2 envelope glycoproteins (data not shown). Therefore, the increased sensitivity of Tier 1 viruses is not associated with an increased propensity to occupy a CD4-bound state nor can it be attributed to global or localized differences in antigenicity. Rather, an increased level of envelope glycoprotein reactivity to the binding of the inhibitors dictates this phenotype.

Although neutralization-resistant Tier 2 HIV-1 are more common *in vivo*, Tier 1 viruses emerge and may predominate in compartments, like the central nervous system, with potential target cells expressing low levels of CD4. HIV-1 with low CD4 requirements for entry are probably counterselected by the humoral immune response. Indeed, passage in monkeys of a simian-human immunodeficiency virus bearing the envelope glycoproteins of the Tier 1 HIV-1 HxBc2 resulted in a neutralization-resistant virus; of note, changes in the gp41 residues 626 and 674, which were identified in our study as determinants of intrinsic reactivity, occurred during the passage and were shown to contribute to neutralization resistance [67].

The magnitude of the effect of changes in intrinsic reactivity on neutralization sensitivity depended upon the inhibitory ligand. This ligand dependency was similarly observed in both the panel of AD8 variants and in the panel of primary HIV-1 isolates, including Tier 1, Tier 2 and transmitted/founder viruses. Thus, antibodies (such as 2G12 and IgG1 b12) that demonstrated low dependence on intrinsic reactivity for inhibition in the panel of AD8 variants also demonstrated a lower dependence on intrinsic reactivity for inhibition within the cohort of primary isolates; neutralization of the primary viruses by these antibodies correlated well with envelope glycoprotein trimer binding. Future studies will investigate the ligand-specific properties that influence the impact of intrinsic reactivity on the susceptibility of HIV-1 to neutralization.

Future studies will also be directed towards identifying better structural and/or phenotypic markers of HIV-1 envelope glycoprotein intrinsic reactivity. Although intrinsically reactive envelope glycoproteins were found to be sensitive to cold exposure and to inhibition by sCD4, neither of these properties were specific indicators of intrinsic reactivity. The S375W gp120 mutant and some Tier 2 HIV-1 were found to be cold-sensitive but not globally neutralization-sensitive, suggesting that pathways to cold sensitivity exist that do not involve increases in intrinsic reactivity (see Figure 10). It is noteworthy that the sensitivity of a range of Tier 1, Tier 2 and transmitted/founder HIV-1 to sCD4 inhibition was predicted only by taking into account both sCD4 binding to the envelope glycoprotein trimer and cold sensitivity. The utility of the latter parameter in strengthening the correlation between sCD4 binding and inhibition supports the relatedness of the envelope glycoprotein changes that predispose to HIV-1 inactivation by cold or sCD4 engagement. Understanding these changes will be helpful in potentiating the HIV-1-inactivating mechanisms of CD4-mimetic small molecules.

The concept of intrinsic envelope glycoprotein reactivity will assist our understanding of primate immunodeficiency virus adaptation to host body compartments, immune responses and antiviral agents.

## Materials and Methods

### Reagents and Antibodies

The IgG1 b12 antibody, which recognizes the CD4-binding site of gp120 [79], [80], and the PG16 antibody, which recognizes a trimer-sensitive epitope dependent on the gp120 V1/V2 and V3 variable loops [50], were kind gifts from Dennis Burton. The F105 antibody, which also recognizes the gp120 CD4-binding site [81], was kindly provided by Marshall Posner [82]. James Robinson kindly provided the following antibodies: 17b and 48d, which recognize gp120 epitopes that are induced by CD4 binding [49], and the anti-gp41 antibodies 7B2 and 2.2B [83]. The 2G12 antibody, which targets a carbohydrate-dependent gp120 epitope [48], and the 2F5 antibody, which recognizes the membrane-proximal external region (MPER) of gp41 [84], were provided by Hermann Katinger. The CD4-binding site-directed antibodies b6 and b13 were provided by Dennis Burton and Peter Kwong [61], [85]. The D49 antibody recognizes the gp41 cluster I immunodominant epitope [86] and was obtained from the International AIDS Vaccine Initiative (IAVI) Neutralizing Antibody Consortium.

The C34 peptide is composed of the HR2 region of the HXBc2 gp41 glycoprotein [amino acid residues 628–661, numbered according to current convention [87] and was custom synthesized by American Peptide Co. The C34-Ig fusion protein consists of the Fc region of human IgG1 linked to two copies of the C34 peptide. C34-Ig was produced and purified as previously described [53]. The CD4-Ig fusion protein consists of the first two N-terminal domains of the CD4 molecule and the Fc region of human IgG1. Purification was carried out as described for the C34-Ig molecule. Four-domain sCD4 was purified from the culture medium of a 293F producer cell line, as previously described [88]. Compound JRC-II-191 is a small-molecule CD4-mimetic inhibitor of HIV-1 [89].

### Envelope Glycoprotein Constructs

The AD8 clone of HIV-1 (accession number AF004394) is a derivative of the macrophage-tropic ADA strain (env accession number M60472). AD8 and ADA envelope glycoproteins were expressed from the pSVIIIenv vector [90]. The vectors were constructed by replacing the *KpnI*(6347)-B*am*HI(8475) fragment of the HXBc2 *env* sequence (accession member K03455) of the original pSVIIIenv plasmid with the corresponding sequences from AD8. Chimeras between AD8 and HXBc2 strains were generated by using the Ale I, Mwo I and Ssp I restriction sites located in the AD8 env genes at nucleotide positions 8044, 8178 and 8276, respectively (numbered according to current convention [87]).

Constructs that contain a deletion of the gp120 V1/V2 variable loops (amino acid residues 128 to 194 deleted) were generated by the overlap PCR method [91] and contain a Gly-Ala-Gly linker in place of the V1/V2 loops [92]. Unless otherwise specified, all envelope glycoproteins with a gp41 cytoplasmic domain deletion contain a 5-amino acid cytoplasmic tail, truncated after Gln 710. The ΔKS construct, which contains an HIV-1 HXBc2 env gene with a large deletion, was used as negative control. Unless indicated otherwise, all envelope glycoproteins described have a complete gp41 cytoplasmic tail.

For studies of HIV-1 sensitivity to cold inactivation, we examined a panel of 16 CCR5-tropic HIV-1 envelope glycoproteins that serve as standards for the evaluation of neutralizing antibody responses to administered vaccines [71], [72], [73], [93]. Tier 1A envelope glycoproteins included: clade A/G DJ263.8; clade B SF162.LS, BAL.26; and clade C MW965.2612. Tier 1B envelope glycoproteins included clade B 6535.3. Tier 2 envelope glycoproteins included clade B QHO692.42, SC422661.8, PVO.4, AC10.029, RHPA4259.7, TRO.11, THRO4156.18, REJO4541.67, TRJO4551.58, WITO4160.33 and CAAN5342.A2, as well as the envelope glycoproteins derived from transmitted/founder subtype B clones: WEAU-d15.410.787, 1006-11.C3.1601, 1054-07.TC4.1499, 1056-10.TA11.1826, 1012-11.TC21.3257, 6240.08.TA5.4622, 6244.13.B5.4576, 62357.14.D3.4589, 9021.12.B2.4571, 700010040.C9.4520, PRB926-04.A9.4237, and SC05.8C11.2344**.** The envelope glycoproteins of isolates Bx08.16 (clade B) and 271-11 (clade A/G) were included in studies of sCD4 binding due to their high sensitivity to this reagent. The GenBank accession numbers of the above panel of envelope glycoproteins are listed in Table 1 of Reference [73].

### Binding of Soluble gp120 to Cells that Express CCR5

Human 293T cells were seeded in 6-well plates (1×10^6^ cells/well) and transfected the next day with plasmids that express the full-length envelope glycoproteins and Tat (0.6 and 0.06 µg/well, respectively) using Effectene transfection reagent (Qiagen). On the following day, culture medium was removed and 2 ml of labeling medium (10% heat-inactivated, dialyzed fetal bovine serum [FBS]; 10 µg/ml penicillin-streptomycin solution; 50 µCi/ml ^35^S-Express protein labeling mix [Perkin Elmer]; and 2 mM L-glutamine in Dulbecco's modified Eagle medium [DMEM]) was added to the transfected cells. Cells were incubated at 37°C for another day before the medium was harvested and cleared of cells and debris by centrifugation (1,000×g for 5 minutes at room temperature) followed by filtration (0.45-µm pore size filter).

For initial quantitation of gp120 shed into the supernatants, serial dilutions of the samples were used for immunoprecipitation by 2.5 µl of pooled serum from HIV-1-infected individuals and Protein A-Sepharose beads in a total volume of 500 µl. After incubation for 2 hours at 4°C, the beads were washed once with DMEM supplemented with 10% FBS (DMEM/10% FBS), three times with NP40 buffer and once with Tris-NaCl buffer (0.15 M NaCl, 10 mM Tris, pH 7.5). Beads were then boiled in sample buffer for 10 minutes. Precipitated proteins were separated by sodium dodecyl sulfate/polyacrylamide gel electrophoresis (SDS-PAGE) and quantified by densitometry.

For CCR5 binding measurements, input gp120 concentrations were equalized according to the above quantitation by dilution with DMEM. To one set of samples, sCD4 was added to a final concentration of 200 nM; the other set of samples was left untreated. All samples were incubated at 37°C for 1 hour and then added to 3×10^6^ Cf2Th cells that stably express human CCR5. The mixtures of supernatant and cells were incubated at 37°C for 2 hours. Cells were then washed three times with DMEM/10% FBS and lysed with NP40 buffer for 30 minutes at 4°C, with gentle agitation. Lysates were cleared by centrifugation at 14,000×g for 30 min at 4°C. CCR5-bound gp120 was precipitated by pooled serum from HIV-1-infected individuals, as described above, and analyzed by SDS-PAGE and densitometry.

### Preparation of Recombinant Luciferase-Expressing Viruses

Single-round, recombinant HIV-1 viruses that express the luciferase gene were generated by transfection of 293T cells using Effectene transfection reagent. Briefly, cells were seeded in 6-well plate wells (approximately 1.5 × 10^6^ cells per well) and transfected the next day with 0.2 µg of the HIV-1 packaging construct pCMVΔP1ΔenvpA, 0.6 µg of the firefly luciferase-expressing construct pHIvec2.luc and 0.2 µg of the plasmid expressing the HIV-1 Rev protein and the envelope glycoproteins, as previously described [47]. On the next day, transfection medium was changed to culture medium (DMEM/10% FBS). Virus-containing supernatants were collected on the following day, cleared of cell debris by low-speed centrifugation and filtered through 0.45-µm filters. Virus particle production was quantified by measuring reverse transcriptase (RT) activity in virus preparations, as previously described [94].

### Infection by Single-Round Luciferase-Expressing Viruses

Target cells were seeded approximately 16 hours before infection at a density of 8×10^3^ cells/well in 96-well luminometer-compatible tissue culture plates (PerkinElmer). For infectivity assays, virus input was normalized for RT unit content and added to the cells in a final volume of 150 µl per well. For neutralization assays, virus was incubated in the presence of the inhibitor for one hr at 37°C. All infections were carried out by incubation of virus with cells for two days at 37°C. For neutralization assays, viruses were incubated with the inhibitor for one hr at 37°C and then added to CD4^+^CCR5^+^ Cf2Th cells. Medium was then removed and cells were lysed with 35 µl of passive lysis buffer (Promega). Cultures were then subjected to three freeze-thaw cycles. To measure luciferase activity, 100 µl of luciferin buffer (15 mM MgSO_4_, 15 mM KPO_4_ [pH 7.8], 1 mM ATP, and 1 mM dithiothreitol) and 50 µl of 1 mM D-luciferin potassium salt (BD Pharmingen) were added to each well. Luminescence was recorded using a Berthold LB 960 microplate luminometer.

### Virus Sensitivity to Cold Inactivation

Recombinant viruses were generated as described above. Each preparation was then diluted and aliquoted, one sample for each prospective time point. All samples were then snap-frozen on dry ice/ethanol for 15 minutes and stored at −80°C. This method of freezing significantly reduced virus inactivation during freezing/thawing. At different time points, the various samples were thawed by incubation in a 37°C water bath for 2 minutes and then placed on ice. After incubation on ice for different lengths of time, virus samples were incubated in a 37°C water bath for 2 minutes and then added to CD4^+^CCR5^+^ Cf2Th cells. Two days later, cells were lysed and assayed for luciferase activity, as described above.

### Virus Capture Assay

To measure cold-induced changes in the capacity of virus particles to bind the 2G12 antibody, we used a virus capture assay [83]. Viruses that express the luciferase gene and contain both AD8 mutant envelope glycoproteins and the Vesicular Stomatitis Virus G glycoprotein (VSV-G) were generated as described above, with the exception that 0.2 µg of a VSV-G expressing plasmid was added to the transfection mix. The monoclonal antibody 2G12 was used to coat protein-binding 96-well plates (Costar) by incubation at 1 µg/ml in PBS for three hours at room temperature. Wells were then washed 3 times with PBS and blocked for two hours with 3% BSA. Virus preparations, which were previously incubated on ice for different time periods were then added to the wells and incubated for three hours at room temperature. All wells were subsequently washed three times with DMEM/10% FBS and then overlaid with CD4^−^CCR5^−^ Cf2Th cells (20,000 cells/well). Cells were incubated overnight at 37°C and then split into luminometer-compatible IsoPlate-96 tissue culture plates (PerkinElmer). On the following day, cells were lysed and luciferase activity measured.

### Measurement of Cold-Induced Shedding of gp120 from Virions

Virus preparations were harvested as described above and centrifuged at 100,000×g for 1 hour at 16°C in a SW-28 rotor (Beckman) over a cushion of 30% sucrose in PBS. Pelleted virions were then resuspended in PBS supplemented with 1% BSA (pH 7.33), aliquoted (100 µl for each time point) and snap-frozen in dry ice immersed in ethanol. A sample of each variant was analyzed by SDS-PAGE and Western blotting to determine the level of envelope glycoprotein incorporation into virions. All other samples were thawed separately and placed on ice for various periods of time. As a control, one sample for each envelope glycoprotein variant was not incubated on ice, but kept frozen. After all samples were thawed, they were centrifuged at 10,750×*g* for 3 hours; the supernatant was then removed. Both pellet and supernatant were separated by SDS-PAGE. Proteins were Western blotted and probed with serum derived from HIV-1-infected individuals.

### Cell-Based Enzyme-Linked Immunosorbent Assay (ELISA)

The binding of antibodies to HIV-1 envelope glycoprotein trimers expressed on cells was measured using a cell-based ELISA system, as previously described [47]. Briefly, COS-1 cells were seeded in 96-well plates (1.8×10^4^ cells/well) and transfected the next day with 0.1 µg of a plasmid expressing the envelope glycoproteins and 0.01 µg of a Tat-expressing plasmid per well using Effectene transfection reagent. Three days later, cells were washed twice with blocking buffer (35 mg/ml BSA, 10 mg/ml non-fat dry milk, 1.8 mM CaCl_2_, 1 mM MgCl_2_, 25 mM Tris, pH 7.5 and 140 mM NaCl) and incubated with the indicated primary antibody for 45 minutes. Cells were then washed four times with blocking buffer and four times with washing buffer (140 mM NaCl, 1.8 mM CaCl2, 1 mM MgCl2 and 20 mM Tris, pH 7.5). A horseradish peroxidase-conjugated antibody specific for the Fc region of human IgG was then incubated with the samples for 45 minutes at room temperature. Cells were washed 5 times with blocking buffer and 5 times with washing buffer. HRP enzyme activity was determined after the addition of 33 µl per well of a 1∶1 mix of Western Lightning oxidizing and luminol reagents (Perkin Elmer Life Sciences) supplemented with 150 mM NaCl. Light emission was measured with a Mithras LB 940 luminometer (Berthold Technologies).

### Measurement of Cell Surface Expression Levels of Envelope Glycoproteins by Immunoprecipitation

COS-1 cells were seeded in 6-well plates and transfected the next day with 0.8 µg of plasmid expressing the envelope glycoproteins and 0.08 µg of Tat-expressing plasmid. Thirty hours after transfection, cells were washed once with cysteine- and methionine-free DMEM. Then 2 ml of labeling medium containing ^35^S-Express protein labeling mix was added (as described for the CCR5-binding assay). Sixteen hours later, cells were detached from the plate using PBS supplemented with 7.5 mM EDTA, washed twice with wash buffer (30 mg/ml BSA in PBS, pH 7.3) and then incubated at 4°C for one hour with an antibody mix containing 2.25 µg/ml of each of the antibodies 2G12, VRC01, 412d and CD4-Ig in wash buffer. Cells were subsequently washed three times with wash buffer and once with PBS and lysed with NP-40 buffer for 30 minutes at 4°C with gentle agitation. Lysates were cleared by centrifugation at 14,000×g for 30 min at 4°C. Antibody-bound gp120 was precipitated using Protein A-Sepharose beads. The protein content in the lysates was measured with the Bradford assay to adjust for input and samples were subsequently analyzed by SDS-PAGE and densitometry. Cell-surface expression levels were defined as the gp120 band intensity subtracted from background signal (mock-transfected cells). Steady-state binding of each inhibitor to the cell surface was measured by cell-based ELISA at 37°C. Inhibitor binding efficiency is defined as the level of inhibitor binding divided by the relative level of cell-surface expression. Binding values (absolute and normalized) that were lower than 1% of the maximal value measured were excluded from the analysis.

### Neutralization-to-Binding Ratios and Average Neutralization-to-Binding Index

The neutralization-to-binding ratio for a given inhibitor was calculated by dividing the neutralization sensitivity of viruses containing the envelope glycoproteins (defined as the reciprocal of the IC50 value) by the normalized binding of the inhibitor to the cell-surface envelope glycoproteins, determined as described above.

The average neutralization-to-binding index was calculated as follows. Based on the neutralization-to-binding ratio, the envelope glycoproteins were arranged in rank order and assigned a rank order value, so that the envelope glycoprotein strain with the highest ratio was assigned a value of 15 and that with the lowest ratio was assigned a value of 1. For some of the tests, specific envelope glycoproteins were eliminated from consideration due to low binding values (<%1 of the maximal value measured in that group). For groups containing less than 15 envelope glycoproteins, the relative rank order values assigned were adjusted to scale of 1 to 15. The assigned rank order values for multiple inhibitors were averaged to obtain the average neutralization-to-binding index.

### Statistical Analyses

To examine the degree of correlation between the different envelope glycoprotein phenotypes, we calculated the Spearman rank-order correlation coefficient (r_s_). This test is well-suited to our purpose, as it makes no assumptions about the distribution of the measured variables. Furthermore, a single outlier data point does not unduly influence the assessment of correlation. Spearman rank-order correlation coefficients range between values of −1 and +1, with values of +1 indicating that both variables increase together and value of −1 indicating that one variable increases as the other variable decreases. An rs value close to zero represents the absence of a relationship between the ranks. A two-tailed P value of less than 0.05 was considered significant. To all correlation diagrams, a regression line was added for demonstrative purposes.

The envelope glycoprotein variants included in each analysis were:

1. For relative CD4-independent infectivity versus relative antibody binding (Figure 2C): AD8, AD8(197), J1Hx, J1Hx(197), J2Hx(197), J3Hx(197), J3Hx(197,HT), J3Hx(197,N) and J3Hx(197,HT,N);
2. For relative CD4-independent infectivity versus relative C34-Ig binding (Figure 3C), for relative CD4-independent infectivity versus inhibitor sensitivity (Figure 4F), and for inhibitor binding versus inhibitor sensitivity (Figure 6): AD8, AD8(197), J1Hx, J1Hx(197), J3Hx(197), J3Hx(197,HT), J3Hx(197,N) and J3Hx(197,HT,N);
3. For relative CD4-independent infectivity versus relative C34-Ig binding for the panel of envelope glycoprotein variants in which deletion of the V1/V2 loops was combined with the gp41 changes (Figure 3D) and for CD4-independent infectivity versus cold sensitivity (Figure 7B): AD8, AD8(ΔV), J1Hx, J1Hx(ΔV), J2Hx(ΔV), J3Hx(ΔV), J3Hx(ΔV,HT), J1Hx(ΔV,N) and J1Hx(ΔV,HT,N);
4. For relative CD4-independent infectivity versus cold sensitivity (Figure 7A): AD8(197), J1Hx(197), J2Hx(197), J3Hx(197), J3Hx(197,HT), J3Hx(197,N) and J3Hx(197,HT,N); and
5. For cold sensitivity versus the sCD4 neutralization-to-binding ratio (Figure 9B) and for sCD4 neutralization sensitivity versus the product of cold sensitivity and CD4-Ig binding (Figure 9C): In addition to the standardized panel of 16 primary HIV-1 envelope glycoproteins, we included eight transmitted/founder strains (WEAU-d15.410.787, 1006-11.C3.1601, 1056-10.TA11.1826, 1012-11.TC21.3257, 6240.08.TA5.4622, 62357.14.D3.4589, 700010040.C9.4520, and PRB926-04.A9.4237)**.** The envelope glycoproteins of isolates Bx08.16 and 271-11 were also included due to their high sensitivity to inhibition by sCD4.

## Supporting Information

Figure S1 **Identification and phenotypic characterization of envelope glycoprotein changes that contribute to the infection of CD4^−^ CCR5^+^ CELLS.** (**A**) Infection of CD4^−^CCR5^+^ and CD4^+^CCR5^+^ Cf2Th cells by luciferase-expressing viruses that contain the indicated envelope glycoproteins (10,000 RT units per sample). In the upper two panels, data are expressed as mean luciferase activity (relative light units, RLU, ± SEM) of three replicate samples. In the bottom left panel, the relative CD4-independent infectivity (infection of CD4^−^CCR5^+^ cells expressed as a percentage of infection of CD4^+^ CCR5^+^ cells measured for each envelope glycoprotein construct) is presented. In the bottom right panel, the percent maximal infection (infection of CD4^−^CCR5^+^ cells mediated by each envelope glycoprotein expressed as a percentage of maximal infection of CD4^−^ CCR5+ cells measured for the virus containing the J1Hx(197) envelope glycoproteins) is shown. This latter measure of CD4 independence, which is unaffected by the efficiency with which the envelope glycoproteins interact with CD4, is shown for comparison because some of the correlation analyses utilized this parameter. (**B**) Cell-surface immunoprecipitation of envelope glycoproteins. COS-1 cells were transfected with the indicated envelope glycoproteins and labeled with ^35^S-cysteine/methionine. Two days after transfection, cells were incubated with the 2G12 antibody (1 µg/ml) in binding buffer (PBS containing 3% BSA) for 30 min at room temperature. Cells were then washed three times with binding buffer and lysed using NP-40 buffer (0.5 M NaCl, 10 mM Tris, pH 7.5 and 0.5% [vol/vol] NP-40). Envelope glycoprotein-2G12 complexes were precipitated with Protein A-Sepharose beads and analyzed by SDS-PAGE. The envelope glycoprotein bands on the gel were detected by PhosphorImager. (**C**) Virion incorporation of envelope glycoproteins. Viruses containing the indicated envelope glycoprotein variants were generated and purified by ultracentrifugation through a 20% sucrose cushion. The control viruses produced in the pcDNA-transfected cells do not contain envelope glycoproteins. Pellets were then resuspended in PBS and virion content determined by reverse transcriptase (RT) activity. Samples were analyzed by SDS-PAGE (gel loading normalized by RT activity) and Western blotted using pooled serum obtained from HIV-1 infected individuals. The positions of the p24 capsid protein and gp120 envelope glycoprotein are indicated. Results are representative of those obtained in two independent experiments.
(TIF)

Figure S2 **Envelope Glycoprotein binding and neutralization by the trimer-specific antibody PG16.** (**A**) Binding of PG16 (0.2 µg/ml) to COS-1 cells expressing the indicated envelope glycoproteins at 4°C in the absence and presence of sCD4 (20 µg/ml). Data are presented as mean percentage of PG16 binding (± SEM) relative to the binding of the 2G12 antibody (2 µg/ml) to each envelope glycoprotein variant; the data were derived from duplicate experiments. (**B**) Neutralization of viruses containing the indicated envelope glycoprotein variants by the PG16 antibody. Residual infection represents the percentage of infection measured after incubation with the indicated concentration of the PG16 antibody relative to that observed in the absence of antibody. The envelope glycoproteins are color coded according to their relative CD4-independent infectivity (see left column in Figure 4A). Data represent the means derived from three replicate samples.
(TIF)

Figure S3 **Infection of CD4^−^CCR5^+^ and CD4^+^CCR5^+^ cells by viruses that contain a deletion of the gp120 V1/V2 loops (ΔV) and/or the gp41 changes.** The mean luciferase activity (± SEM) from an experiment performed with three replicate samples is presented (37,500 RT units added per well). In the bottom panel, infection of CD4^−^CCR5^+^ cells is expressed as the percentage of infection of CD4^+^CCR5^+^ cells.
(TIF)

Figure S4 **Exposure of the gp41 HR1 coiled coil on the HIV-1 envelope glycoproteins.** (**A**) Effect of envelope glycoprotein cytoplasmic tail deletion on infection of CD4^−^CCR5^+^ and CD4^+^CCR5^+^ cells. Viruses that express the luciferase gene and contain the indicated full-length or cytoplasmic tail-deleted (Δct) envelope glycoproteins were generated and RT activity measured. Virus preparations were incubated with CD4^−^CCR5^+^ or CD4^+^CCR5^+^ cells (10,000 RT units per well). The mean luciferase activity (± SEM) from an experiment performed with three replicate samples is presented. (**B**) Effect of temperature on binding of C34-Ig to cell surface-expressed envelope glycoproteins. COS-1 cells transiently expressing the indicated envelope glycoproteins were incubated with sCD4 (40 µg/ml) or buffer for 3 min at 37°C. Samples were subsequently washed three times to remove excess sCD4 and then incubated with C34-Ig (40 µg/ml) for 30 min at the indicated temperature. Binding of C34-Ig to the envelope glycoproteins is shown in the top panel. Binding of the CD4-Ig probe (0.6 µg/ml) after incubation for 30 min at the indicated temperature is shown in the bottom panel. Data represent the means (± SEM) derived from duplicate samples. (**C**) Binding of C34-Ig to V1/V2 loop-deleted envelope glycoproteins in the absence and presence of sCD4. COS-1 cells that transiently express the indicated cytoplasmic tail-deleted envelope glycoproteins were incubated with C34-Ig (30 µg/ml) at 26°C for 30 min in the absence or presence of sCD4 (20 µg/ml). Data represent mean (± SEM) binding values after subtraction of background values of C34-Ig binding to mock-transfected cells. In the bottom panel, C34-Ig binding in the absence of sCD4 is expressed as the percentage of C34-Ig binding in the presence of sCD4. (**D**) Binding of C34-Ig (40 µg/ml) to COS-1 cells expressing the indicated cytoplasmic tail-deleted envelope glycoprotein variants at 26°C, in the absence or presence of sCD4 (20 µg/ml). Binding of CD4-Ig (0.5 µg/ml) was also measured (bottom panel).
(TIF)

Figure S5 **Effect of envelope glycoprotein changes on exposure and stability of the HR1 coiled coil.** (**A**) Effect of the H66N and S375W changes (indicated by 66 and 375, respectively) on the capacity of envelope glycoproteins to infect CD4^−^CCR5^+^ and CD4^+^CCR5^+^ cells (25,000 RT units per well). (**B**) Effect of the H66N and S375W changes on binding of C34-Ig (30 µg/ml) to COS-1 cells expressing the indicated cytoplasmic tail-deleted envelope glycoprotein variants at 16°C, in the absence or presence of sCD4 (20 µg/ml). The deletion of the gp120 V1/V2 variable loops is indicated by ΔV. (**C**) Decay rate of exposure of the gp41 HR1 coiled coil after pulse activation by sCD4. COS-1 cells transiently expressing the indicated envelope glycoprotein variants were incubated with sCD4 (30 µg/ml) for 3 min at 37°C. Cells were then washed to remove excess sCD4 and incubated for different time periods at 37°C. The C34-Ig probe (30 µg/ml) was subsequently incubated with the cells at 25°C. Data represent mean (± SEM) percentages of C34-Ig binding, relative to the C34-Ig binding observed when C34-Ig was added immediately after the sCD4 pulse and subsequent wash.
(TIF)

Figure S6 **Inhibitor sensitivity of viruses containing envelope glycoprotein variants with the N197S and gp41 changes.** Residual infection represents the percentage of infection measured following incubation of the viruses containing the indicated envelope glycoproteins with the inhibitor, relative to that seen in the absence of inhibitor. Color coding in A–F is based on the relative CD4-independent infectivity of viruses with the indicated envelope glycoproteins (see left column in Figure 4A).
(TIF)

Figure S7 **Neutralization sensitivity of V1/V2 loop-deleted envelope glycoprotein variants.** (**A**) Relative CD4-independent infectivity and neutralization sensitivity of viruses that contain the indicated envelope glycoprotein variants. The envelope glycoproteins are color coded for each phenotype as in Figure 4A. (**B–D**) Neutralization of viruses with the indicated envelope glycoproteins by sCD4 and antibodies. Each envelope glycoprotein variant is color coded according to its relative CD4-independent infectivity (left column, panel A).
(TIF)

Figure S8 **Effect of the H66N and S375W changes on CD4 independence and neutralization sensitivity.** (**A**) Infection of CD4^−^CCR5^+^ or CD4^+^CCR5^+^ cells by viruses containing the indicated envelope glycoprotein variants (37,500 RT units per well). (**B–G**) Neutralization sensitivity of viruses containing the indicated envelope glycoproteins. Residual infection represents the percentage of infection measured following incubation of the viruses containing the indicated envelope glycoproteins with the inhibitor, relative to that seen in the absence of inhibitor. Color coding is based on the relative CD4-independent infectivity of each variant (see panel A and Figure 5).
(TIF)

Figure S9 **Ligand binding to cell surface-expressed envelope glycoproteins.** (**A**) Binding kinetics of 17b antibody to envelope glycoprotein trimers expressed on the surface of cells. COS-1 cells expressing the indicated envelope glycoprotein variants were incubated with the 17b antibody (2 µg/ml) at 37°C for different time periods. Cells were then washed to remove excess antibody and binding detected using a secondary HRP-conjugated antibody. Data are presented as a percentage of maximal binding measured at the last time point. Envelope glycoproteins are color coded according to their sensitivity to neutralization by 17b. Absolute binding values measured at the last time point are shown in the panel on the right. (**B**) Effect of inhibitor binding to envelope glycoprotein trimers on recognition by the 412d antibody. COS-1 cells expressing the indicated envelope glycoprotein variants were incubated for 30 min at 37°C with JRC-II-191 (200 µM), sCD4 (400 nM), the F105 antibody (10 µg/ml), sCD4 and C34 peptide (400 nM and 1 µM, respectively) or the D49 antibody (10 µg/ml). Cells were then washed three times and incubated with the 412d antibody (0.7 µg/ml). 412d binding was detected using a preparation of HRP-conjugated goat-anti-human IgG secondary antibodies that does not bind to the F105 antibody or to the mouse D49 antibody. Data represent mean binding values (± SEM) derived from duplicate samples. As expected, initial incubation with sCD4 or the CD4-mimetic compound JRC-II-191 increased 412d binding, whereas pre-incubation with the F105 antibody significantly decreased 412d binding, as previously shown (references 45,84). (**C**) Effect of inhibitor binding to envelope glycoprotein trimers on recognition by 2F5, sCD4 and 2G12. COS-1 cells expressing the indicated envelope glycoprotein variants were incubated with the priming antibodies F105, 17b, 2F5 or 2G12 (all at 5 µg/ml) or with sCD4 (20 µg/ml) for 30 min at 37°C. Cells were then washed three times and incubated with sCD4 (left panel, 10 µg/ml), 2F5 antibody (middle panel, 1 µg/ml) or the 2G12 antibody (right panel, 1 µg/ml). Binding of sCD4 was detected using the OKT4 antibody (10 µg/ml). Binding of 2F5 and 2G12 was detected using a preparation of HRP-conjugated goat-anti-human IgG secondary antibodies that does not bind to the F105 antibody. Data represent mean binding values (± SEM) derived from duplicate samples. The finding that exposure to the inhibitor does not result in altered antigenicity of the envelope glycoproteins suggests that inhibition is not mediated by increased gp120 shedding,
(TIF)

Figure S10 **CD4 independence and cold sensitivity of envelope glycoproteins.** (**A**) Sensitivity of the indicated envelope glycoprotein variants to cold-induced inactivation. The level of infection of CD4^+^CCR5^+^ cells by viruses with the indicated envelope glycoproteins following incubation on ice for the times shown was divided by the level of infection seen for freshly thawed viruses to yield the residual infection. Data represent mean levels of infection (± SEM) derived from three replicate samples. (**B**) Effect of the S375W change in gp120 on cold sensitivity of viruses containing the indicated envelope glycoproteins. Cold sensitivity was measured as in (**A**). Data represent mean percentages of residual infection (± SEM) derived from three replicate samples. (**C**) Effect of the CD4-mimetic compound JRC-II-191 on cold sensitivity. Viruses containing the indicated envelope glycoprotein variants were incubated for different time periods on ice in the absence or presence of JRC-II-191 (20 µM). Samples were then added to CD4^+^CCR5^+^ cells and infectivity was measured 2 days later. Residual infection represents the level of infection, relative to that observed for viruses not incubated on ice. (**D**) Comparison of the cold sensitivity of viruses containing full-length and cytoplasmic tail-deleted (Δct) envelope glycoproteins. In the left column, the relative level of CD4-independent infectivity of the full-length variants is indicated. Values are color coded according to the rank order of the measured phenotype. (**E**) Virus capture assay to measure the presence of gp120 on virions. Viruses that contain both the indicated full-length envelope glycoprotein variants and the vesicular stomatitis virus G protein were incubated for different time periods on ice and then captured on a plate coated with the 2G12 antibody. The amount of virus captured was assessed by measuring infectivity on CD4^−^CCR5^−^ Cf2Th cells. A detailed description of the method is provided in the Materials and Methods. Data represent the mean values (± SEM) derived from three replicate samples. The envelope glycoprotein variants are color coded according to their relative level of cold sensitivity. (**F**) Cold-induced shedding of gp120. Purified virus preparations containing the indicated envelope glycoproteins were incubated for different lengths of time on ice and then virions were pelleted again. Proteins in the pellet and supernatant were separated by SDS-PAGE and Western blotted. The Western blots were probed with serum derived from HIV-1-infected individuals and quantitated by densitometry. Data represent the intensity of the gp120 band in the supernatant at each time point, relative to that measured for samples not incubated on ice. The amount of gp120 in samples not incubated on ice, corrected for virion content by RT activity, is shown in the bottom panel. No significant differences were observed in the gp120 content of the pelleted virions (data not shown). The envelope glycoproteins are color coded according to their relative level of cold sensitivity.
(TIF)

Figure S11 **Neutralization sensitivity of a standardized panel of 16 envelope glycoproteins from primary HIV-1 isolates.** Data were obtained from a registry of molecularly cloned HIV-1, SIV and SHIV envelope glycoproteins that can be used to pseudotype HIV-1 in neutralizing antibody assays; the registry was compiled by the Laboratory for AIDS Vaccine Research & Development, Duke University Medical Center and is available at http://www.hiv.lanl.gov/content/nab-reference-strains/html/home.htm). Data are presented as the concentration of inhibitor at which virus infectivity was reduced by 50% relative to cultures without added inhibitor. The two-tailed T-test P values for the comparison between the IC_50_ values for Tier 1 and Tier 2 envelope glycoproteins are indicated.
(TIF)

Figure S12 **The neutralization-to-Binding Ratio as a measure of envelope glycoprotein reactivity to inhibitor binding.** (**A–D**) Neutralization-to binding ratios for the four inhibitors. Binding of the inhibitors to cell surface-expressed envelope glycoproteins was measured by cell-based ELISA and normalized for cell-surface expression measured by cell-surface immunoprecipitation. Data are expressed as the reciprocal of the IC_50_ value of each envelope glycoprotein strain divided by the normalized binding efficiency. The envelope glycoproteins are colored according to neutralization sensitivity (Tier 1A, black; Tier 1B, grey; and Tier 2, empty bars). The P value for the two-tailed T-Test performed to compare the neutralization-to-binding ratios between Tier 1 and Tier 2 envelope glycoproteins is shown.
(TIF)

Figure S13 **Relationship between cold sensitivity and inhibitor reactivity.** (**A**) The relationship between the cold sensitivity of viruses containing envelope glycoproteins from the standardized panel of 16 primary HIV-1 isolates and the average neutralization-to-binding index for the four inhibitors (see Figure 8B) is shown. (**B–E**) The relationship between cold sensitivity and reactivity to the binding of the indicated inhibitors is shown. The neutralization-to-binding ratio is defined as the reciprocal of the IC50 value of virus containing that envelope glycoprotein divided by the normalized binding efficiency of the inhibitor to the cell surface-expressed envelope glycoproteins. The Spearman rank-order correlation coefficient is indicated (*, P<0.05).
(TIF)

Table S1 **Effect of cytoplasmic tail deletion on binding of CD4-induced antibodies to the envelope glycoproteins.**
(TIF)
