# Supplementary figures and images for: Contribution of Intrinsic Reactivity of the HIV-1 Envelope Glycoproteins to CD4-Independent Infection and Global Inhibitor Sensitivity
Source: PLoS Pathog. 2011 Jun 23;7(6):e1002101. doi: 10.1371/journal.ppat.1002101 (PMC3121797; doi:10.1371/journal.ppat.1002101)

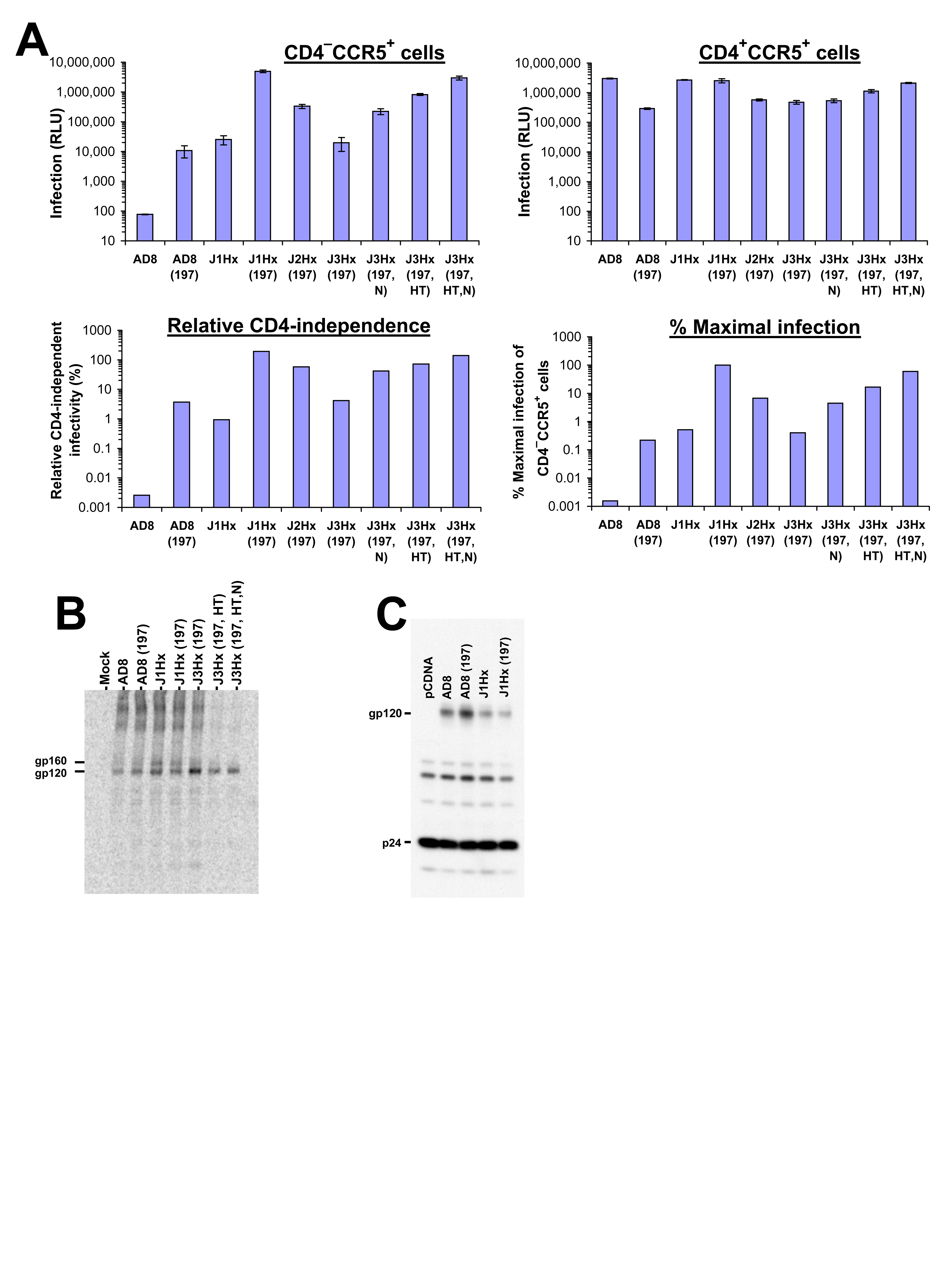

Supplement: Figure S1 — Identification and phenotypic characterization of envelope glycoprotein changes that contribute to the infection of CD4− CCR5+ CELLS. (A) Infection of CD4−CCR5+ and CD4+CCR5+ Cf2Th cells by luciferase-expressing viruses that contain the indicated envelope glycoproteins (10,000 RT units per sample). In the upper two panels, data are expressed as mean luciferase activity (relative light units, RLU, ± SEM) of three replicate samples. In the bottom left panel, the relative CD4-independent infectivity (infection of CD4−CCR5+ cells expressed as a percentage of infection of CD4+ CCR5+ cells measured for each envelope glycoprotein construct) is presented. In the bottom right panel, the percent maximal infection (infection of CD4−CCR5+ cells mediated by each envelope glycoprotein expressed as a percentage of maximal infection of CD4− CCR5+ cells measured for the virus containing the J1Hx(197) envelope glycoproteins) is shown. This latter measure of CD4 independence, which is unaffected by the efficiency with which the envelope glycoproteins interact with CD4, is shown for comparison because some of the correlation analyses utilized this parameter. (B) Cell-surface immunoprecipitation of envelope glycoproteins. COS-1 cells were transfected with the indicated envelope glycoproteins and labeled with 35S-cysteine/methionine. Two days after transfection, cells were incubated with the 2G12 antibody (1 µg/ml) in binding buffer (PBS containing 3% BSA) for 30 min at room temperature. Cells were then washed three times with binding buffer and lysed using NP-40 buffer (0.5 M NaCl, 10 mM Tris, pH 7.5 and 0.5% [vol/vol] NP-40). Envelope glycoprotein-2G12 complexes were precipitated with Protein A-Sepharose beads and analyzed by SDS-PAGE. The envelope glycoprotein bands on the gel were detected by PhosphorImager. (C) Virion incorporation of envelope glycoproteins. Viruses containing the indicated envelope glycoprotein variants were generated and purified by ultracentrifugation through a 2 [file ppat.1002101.s001.tif]

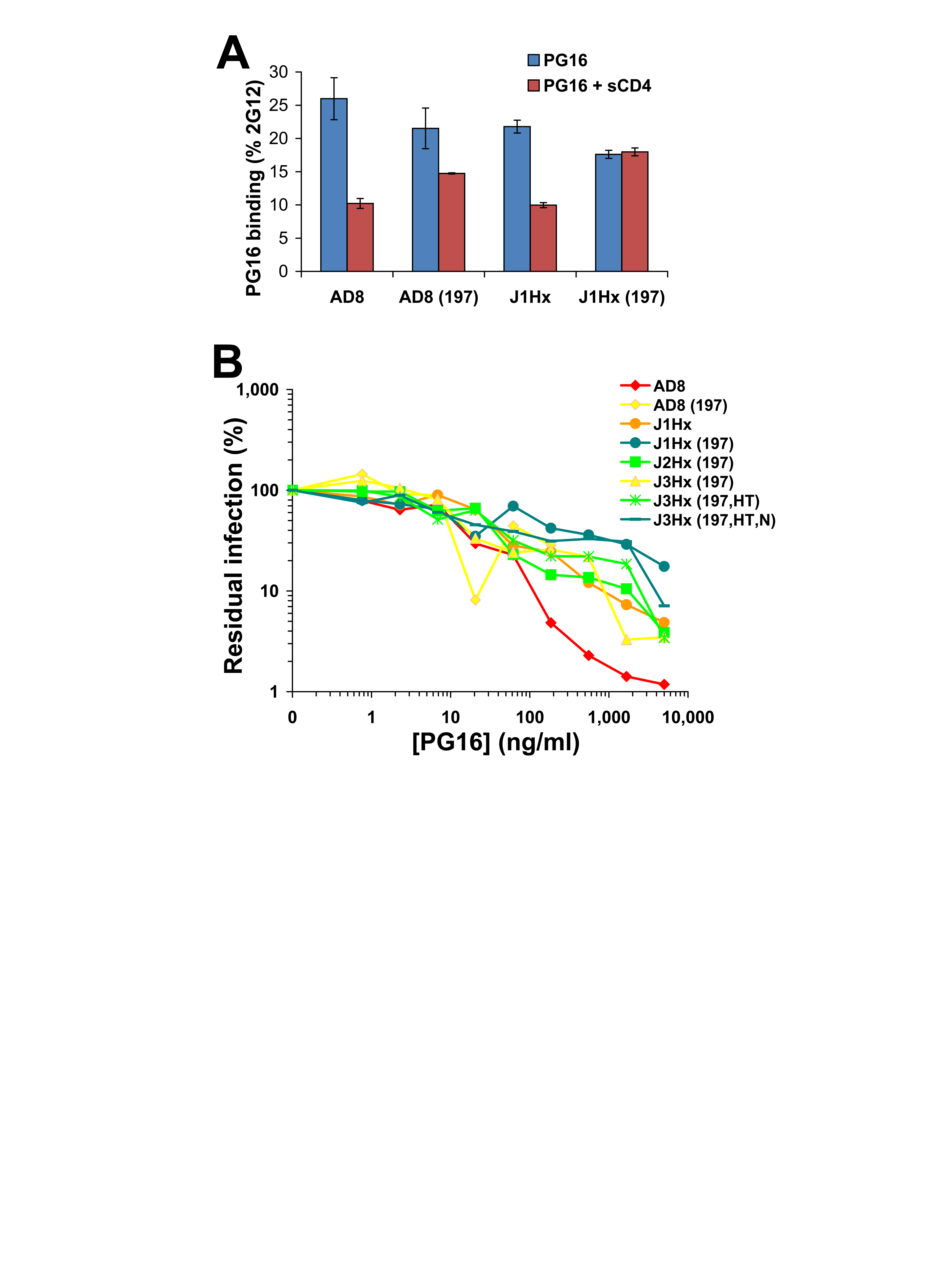

Supplement: Figure S2 — Envelope Glycoprotein binding and neutralization by the trimer-specific antibody PG16. (A) Binding of PG16 (0.2 µg/ml) to COS-1 cells expressing the indicated envelope glycoproteins at 4°C in the absence and presence of sCD4 (20 µg/ml). Data are presented as mean percentage of PG16 binding (± SEM) relative to the binding of the 2G12 antibody (2 µg/ml) to each envelope glycoprotein variant; the data were derived from duplicate experiments. (B) Neutralization of viruses containing the indicated envelope glycoprotein variants by the PG16 antibody. Residual infection represents the percentage of infection measured after incubation with the indicated concentration of the PG16 antibody relative to that observed in the absence of antibody. The envelope glycoproteins are color coded according to their relative CD4-independent infectivity (see left column in Figure 4A). Data represent the means derived from three replicate samples. (TIF) [file ppat.1002101.s002.tif]

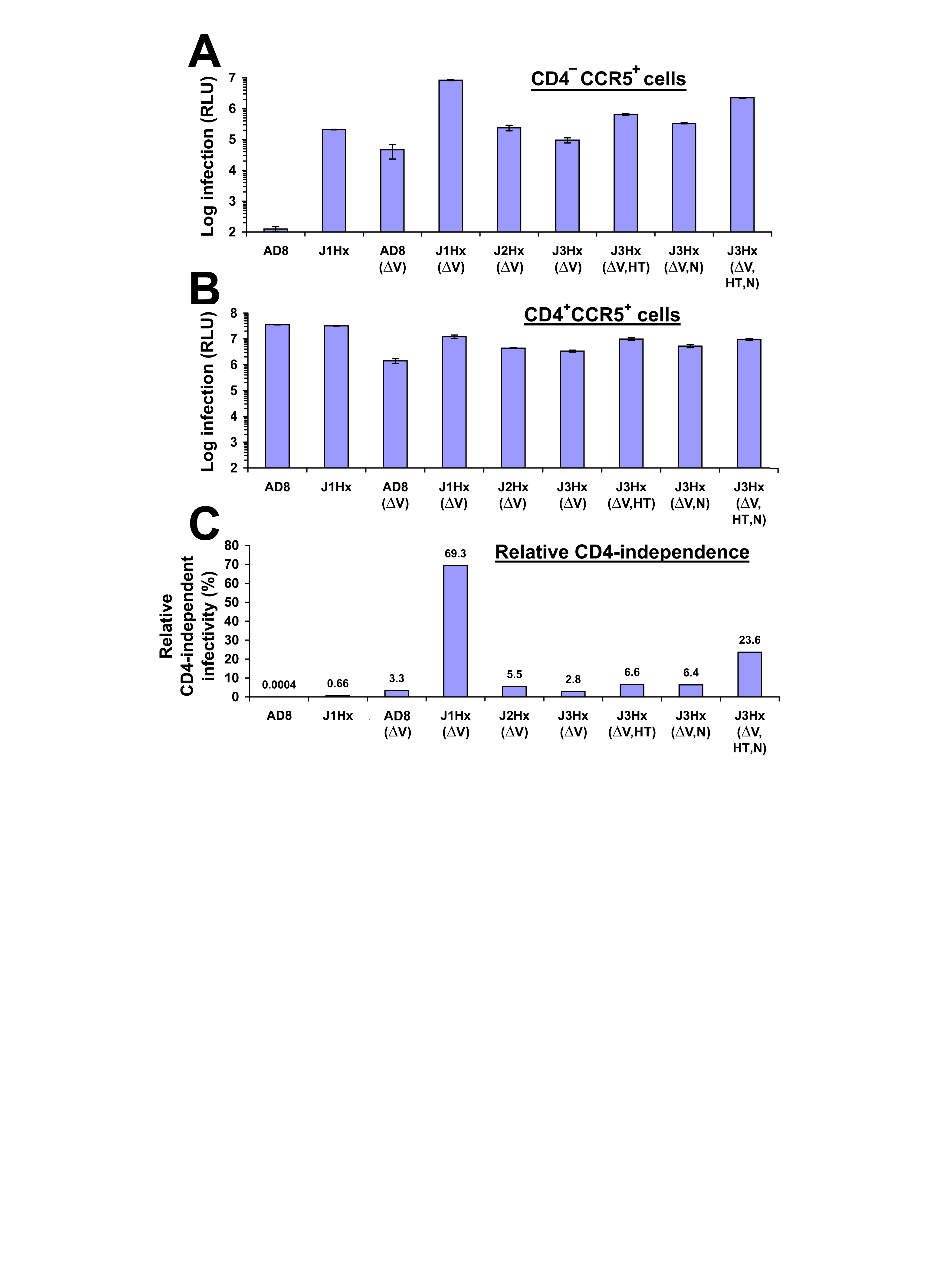

Supplement: Figure S3 — Infection of CD4−CCR5+ and CD4+CCR5+ cells by viruses that contain a deletion of the gp120 V1/V2 loops (ΔV) and/or the gp41 changes. The mean luciferase activity (± SEM) from an experiment performed with three replicate samples is presented (37,500 RT units added per well). In the bottom panel, infection of CD4−CCR5+ cells is expressed as the percentage of infection of CD4+CCR5+ cells. (TIF) [file ppat.1002101.s003.tif]

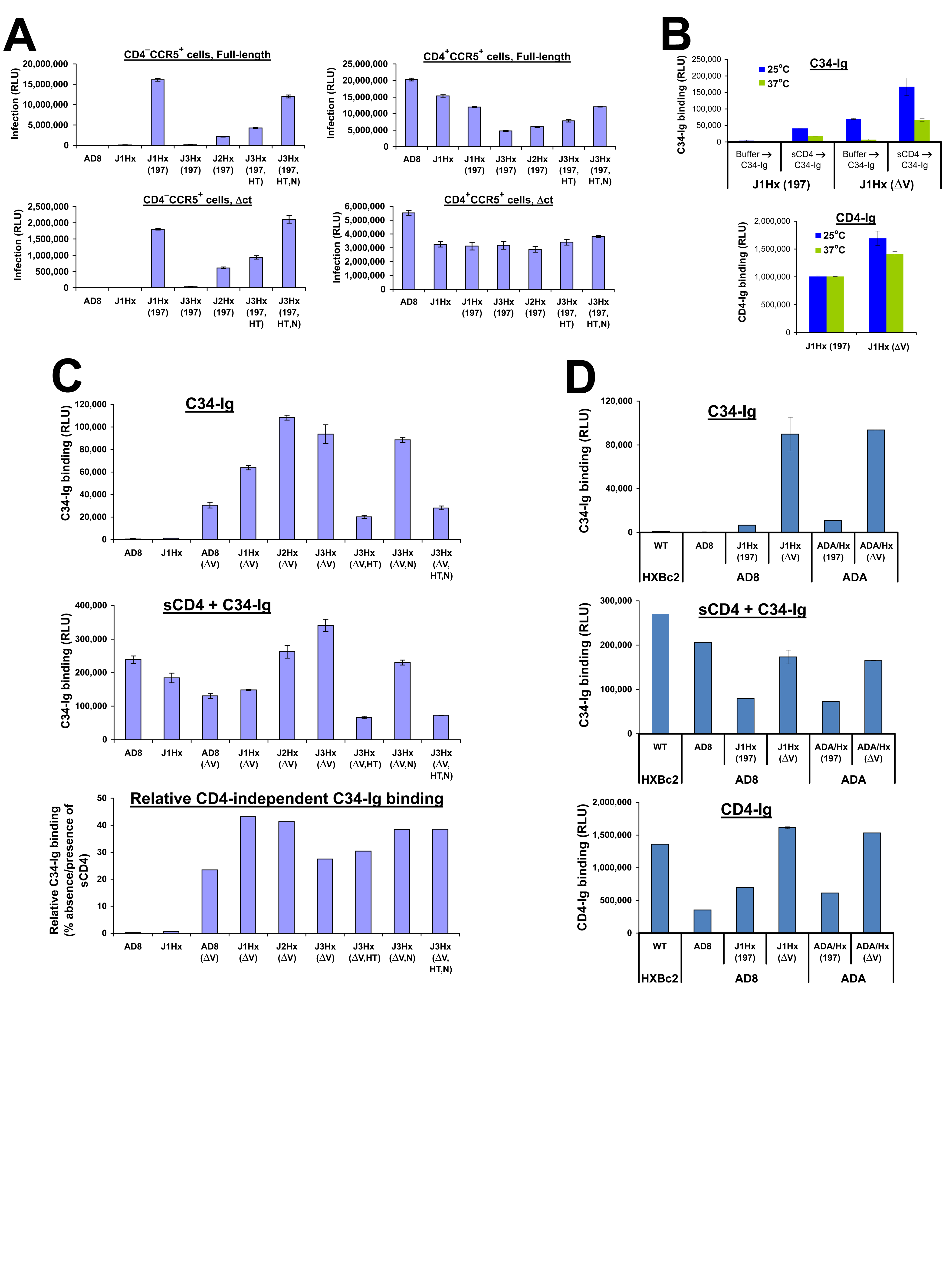

Supplement: Figure S4 — Exposure of the gp41 HR1 coiled coil on the HIV-1 envelope glycoproteins. (A) Effect of envelope glycoprotein cytoplasmic tail deletion on infection of CD4−CCR5+ and CD4+CCR5+ cells. Viruses that express the luciferase gene and contain the indicated full-length or cytoplasmic tail-deleted (Δct) envelope glycoproteins were generated and RT activity measured. Virus preparations were incubated with CD4−CCR5+ or CD4+CCR5+ cells (10,000 RT units per well). The mean luciferase activity (± SEM) from an experiment performed with three replicate samples is presented. (B) Effect of temperature on binding of C34-Ig to cell surface-expressed envelope glycoproteins. COS-1 cells transiently expressing the indicated envelope glycoproteins were incubated with sCD4 (40 µg/ml) or buffer for 3 min at 37°C. Samples were subsequently washed three times to remove excess sCD4 and then incubated with C34-Ig (40 µg/ml) for 30 min at the indicated temperature. Binding of C34-Ig to the envelope glycoproteins is shown in the top panel. Binding of the CD4-Ig probe (0.6 µg/ml) after incubation for 30 min at the indicated temperature is shown in the bottom panel. Data represent the means (± SEM) derived from duplicate samples. (C) Binding of C34-Ig to V1/V2 loop-deleted envelope glycoproteins in the absence and presence of sCD4. COS-1 cells that transiently express the indicated cytoplasmic tail-deleted envelope glycoproteins were incubated with C34-Ig (30 µg/ml) at 26°C for 30 min in the absence or presence of sCD4 (20 µg/ml). Data represent mean (± SEM) binding values after subtraction of background values of C34-Ig binding to mock-transfected cells. In the bottom panel, C34-Ig binding in the absence of sCD4 is expressed as the percentage of C34-Ig binding in the presence of sCD4. (D) Binding of C34-Ig (40 µg/ml) to COS-1 cells expressing the indicated cytoplasmic tail-deleted envelope glycoprotein variants at 26°C, in the absence or presence of sCD4 (20 µg/ml). Binding of CD4-Ig (0.5 µg/ml) wa [file ppat.1002101.s004.tif]

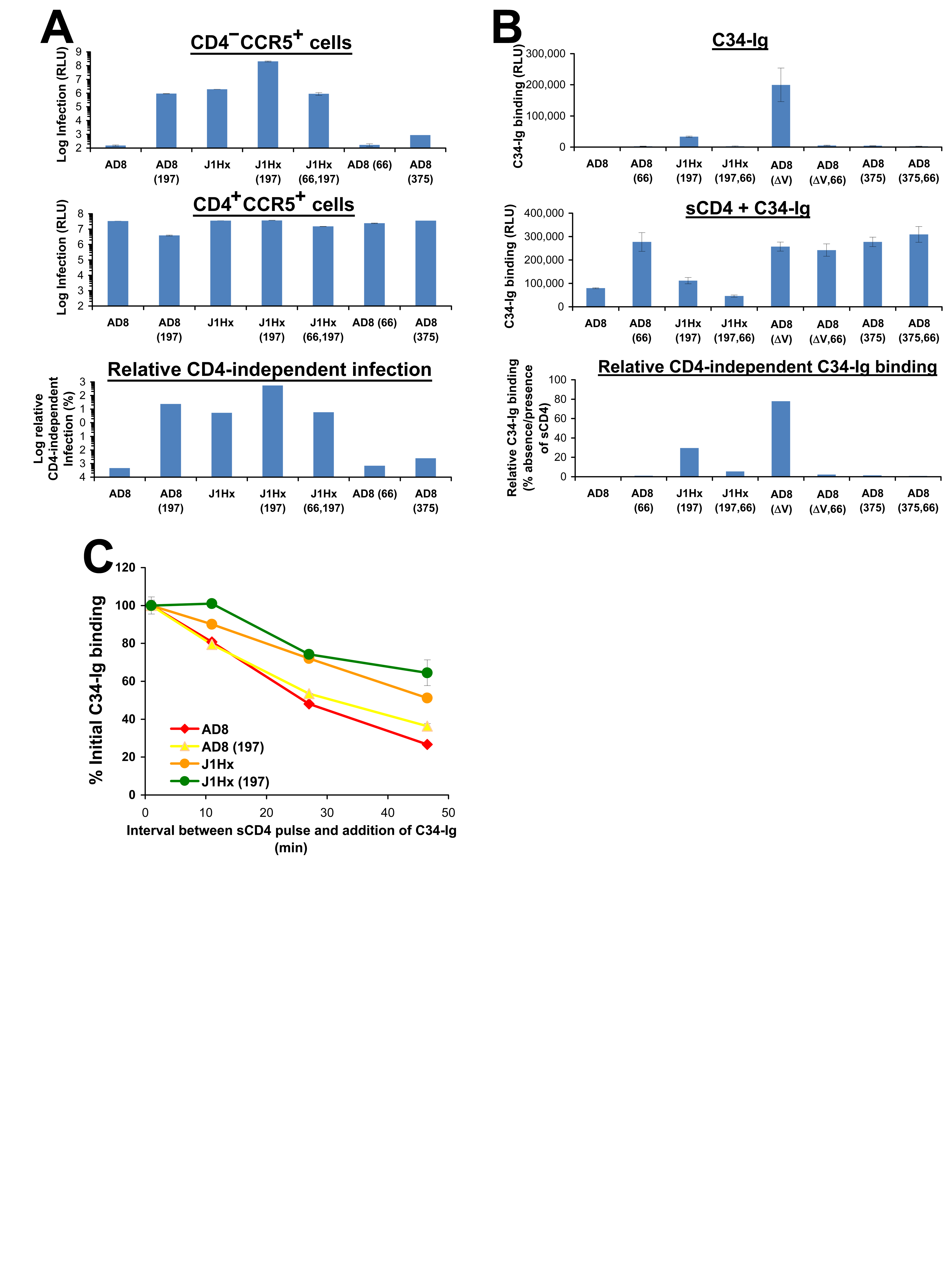

Supplement: Figure S5 — Effect of envelope glycoprotein changes on exposure and stability of the HR1 coiled coil. (A) Effect of the H66N and S375W changes (indicated by 66 and 375, respectively) on the capacity of envelope glycoproteins to infect CD4−CCR5+ and CD4+CCR5+ cells (25,000 RT units per well). (B) Effect of the H66N and S375W changes on binding of C34-Ig (30 µg/ml) to COS-1 cells expressing the indicated cytoplasmic tail-deleted envelope glycoprotein variants at 16°C, in the absence or presence of sCD4 (20 µg/ml). The deletion of the gp120 V1/V2 variable loops is indicated by ΔV. (C) Decay rate of exposure of the gp41 HR1 coiled coil after pulse activation by sCD4. COS-1 cells transiently expressing the indicated envelope glycoprotein variants were incubated with sCD4 (30 µg/ml) for 3 min at 37°C. Cells were then washed to remove excess sCD4 and incubated for different time periods at 37°C. The C34-Ig probe (30 µg/ml) was subsequently incubated with the cells at 25°C. Data represent mean (± SEM) percentages of C34-Ig binding, relative to the C34-Ig binding observed when C34-Ig was added immediately after the sCD4 pulse and subsequent wash. (TIF) [file ppat.1002101.s005.tif]

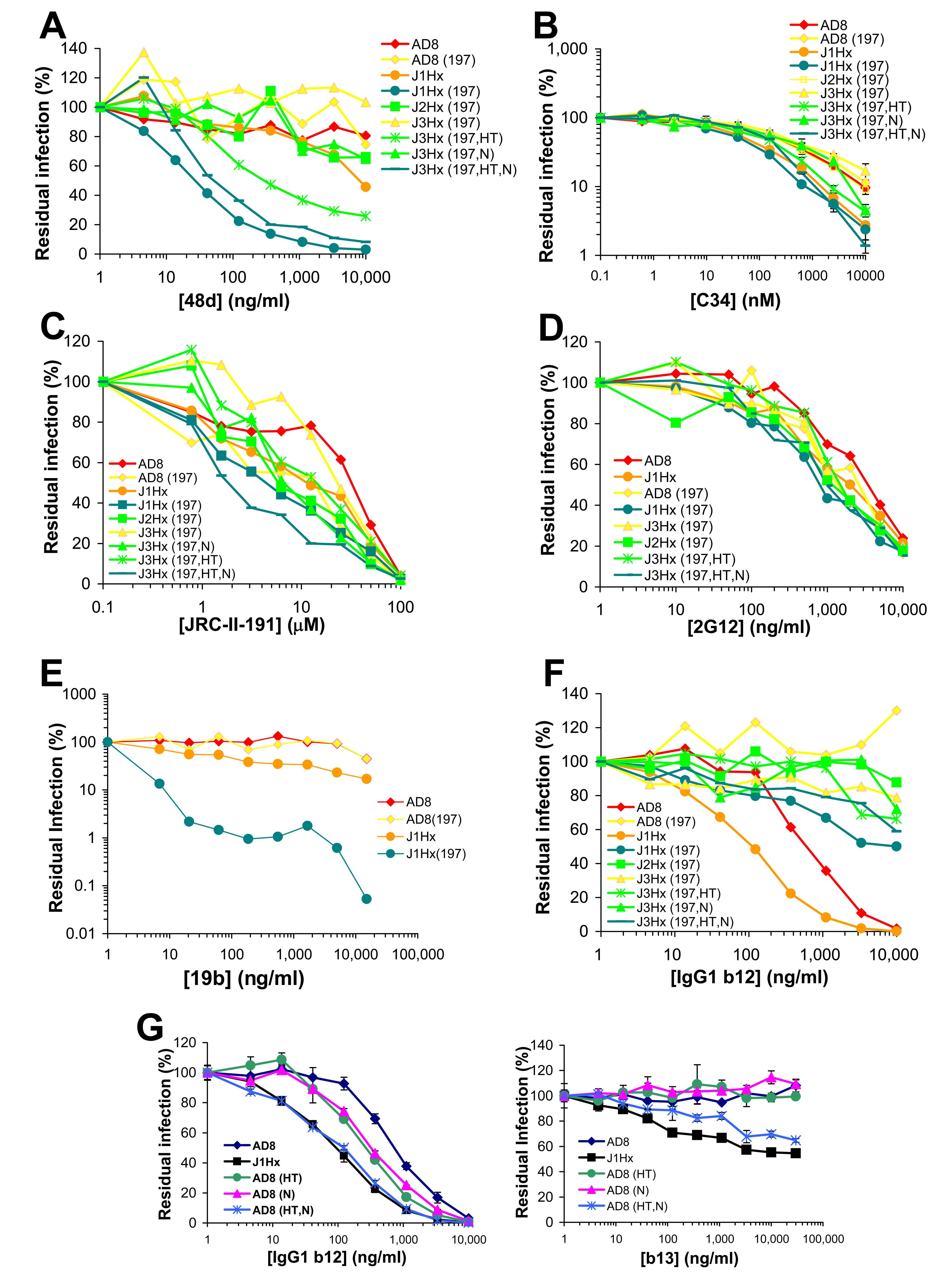

Supplement: Figure S6 — Inhibitor sensitivity of viruses containing envelope glycoprotein variants with the N197S and gp41 changes. Residual infection represents the percentage of infection measured following incubation of the viruses containing the indicated envelope glycoproteins with the inhibitor, relative to that seen in the absence of inhibitor. Color coding in A–F is based on the relative CD4-independent infectivity of viruses with the indicated envelope glycoproteins (see left column in Figure 4A). (TIF) [file ppat.1002101.s006.tif]

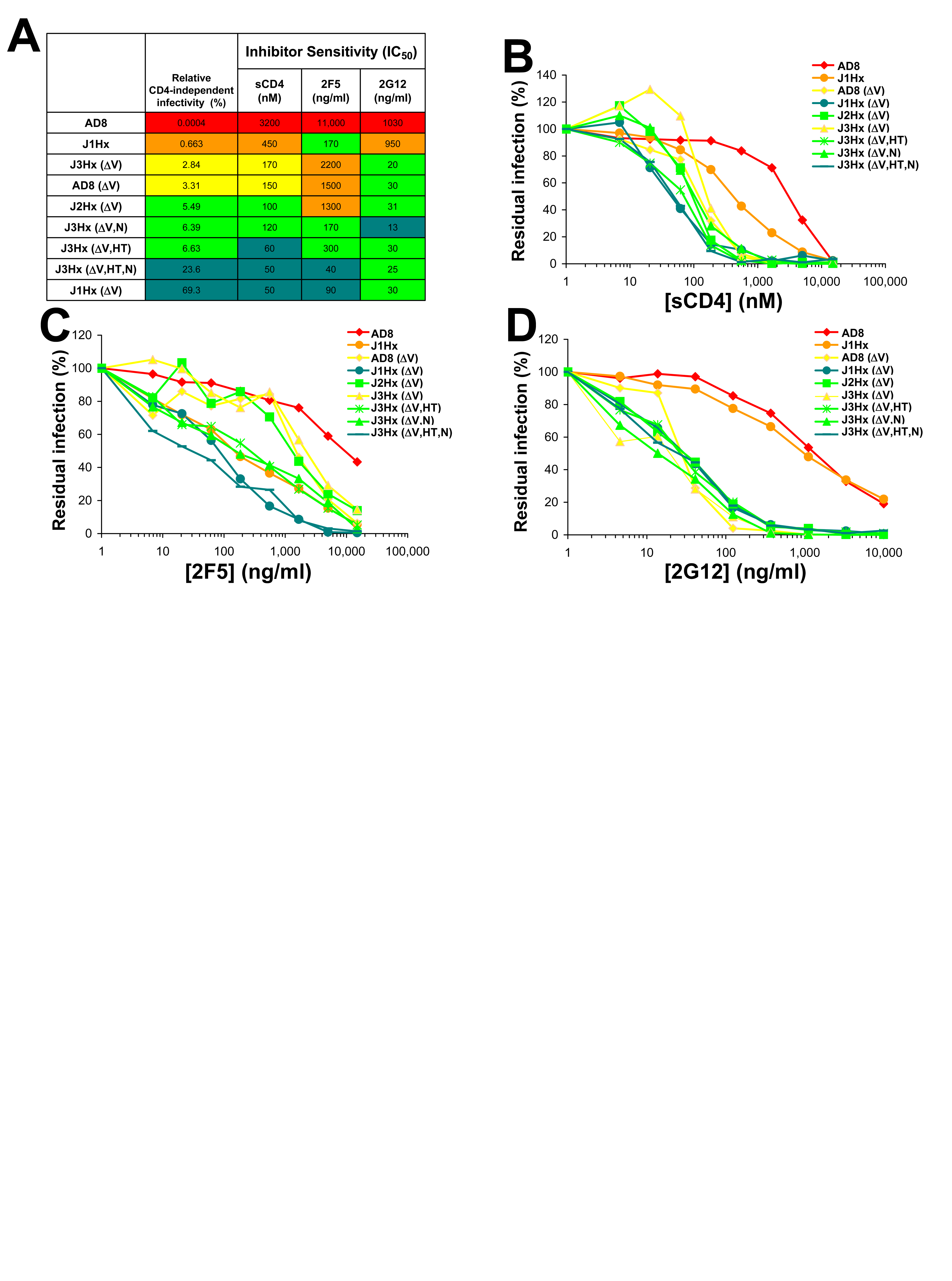

Supplement: Figure S7 — Neutralization sensitivity of V1/V2 loop-deleted envelope glycoprotein variants. (A) Relative CD4-independent infectivity and neutralization sensitivity of viruses that contain the indicated envelope glycoprotein variants. The envelope glycoproteins are color coded for each phenotype as in Figure 4A. (B–D) Neutralization of viruses with the indicated envelope glycoproteins by sCD4 and antibodies. Each envelope glycoprotein variant is color coded according to its relative CD4-independent infectivity (left column, panel A). (TIF) [file ppat.1002101.s007.tif]

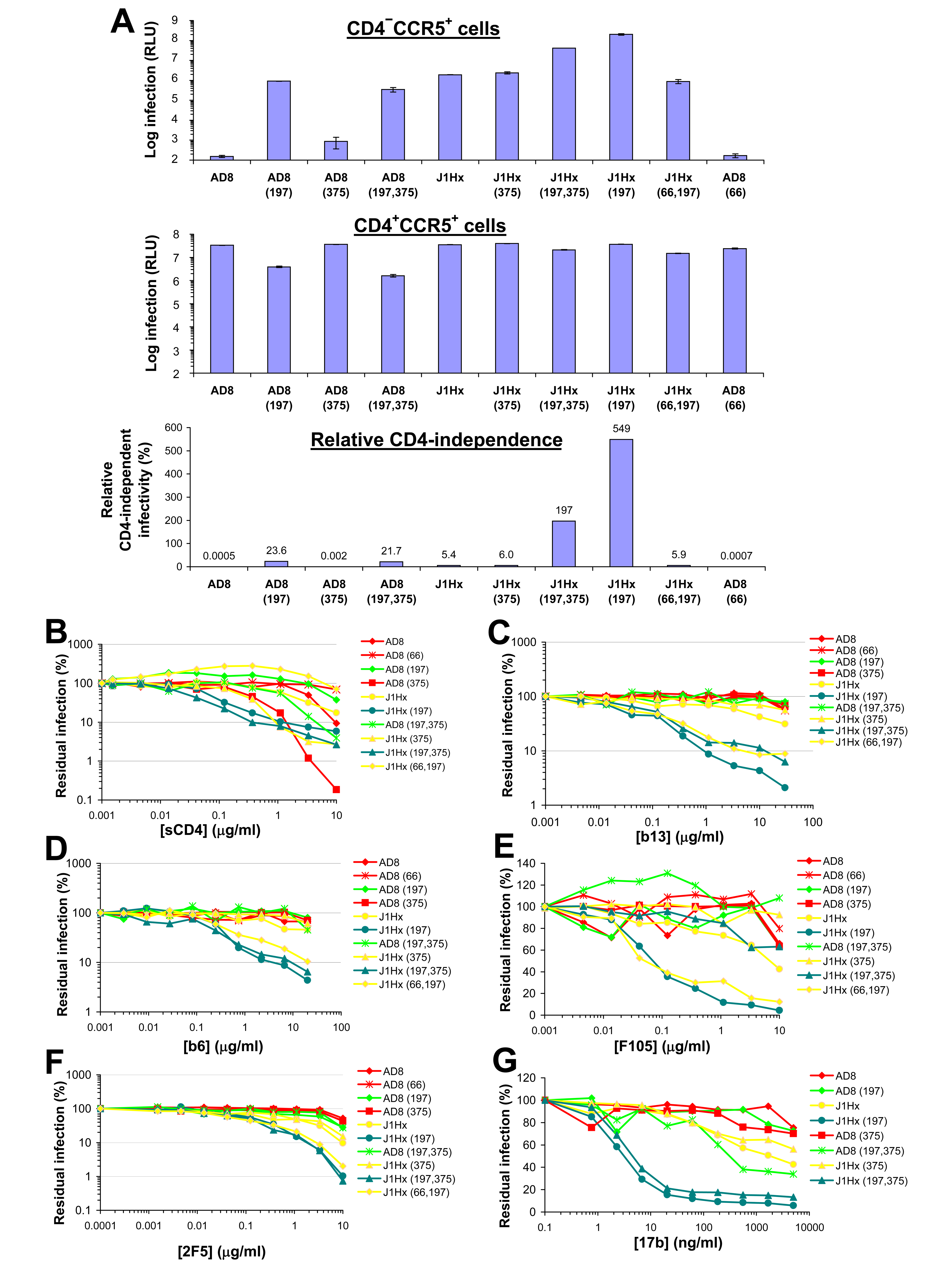

Supplement: Figure S8 — Effect of the H66N and S375W changes on CD4 independence and neutralization sensitivity. (A) Infection of CD4−CCR5+ or CD4+CCR5+ cells by viruses containing the indicated envelope glycoprotein variants (37,500 RT units per well). (B–G) Neutralization sensitivity of viruses containing the indicated envelope glycoproteins. Residual infection represents the percentage of infection measured following incubation of the viruses containing the indicated envelope glycoproteins with the inhibitor, relative to that seen in the absence of inhibitor. Color coding is based on the relative CD4-independent infectivity of each variant (see panel A and Figure 5). (TIF) [file ppat.1002101.s008.tif]

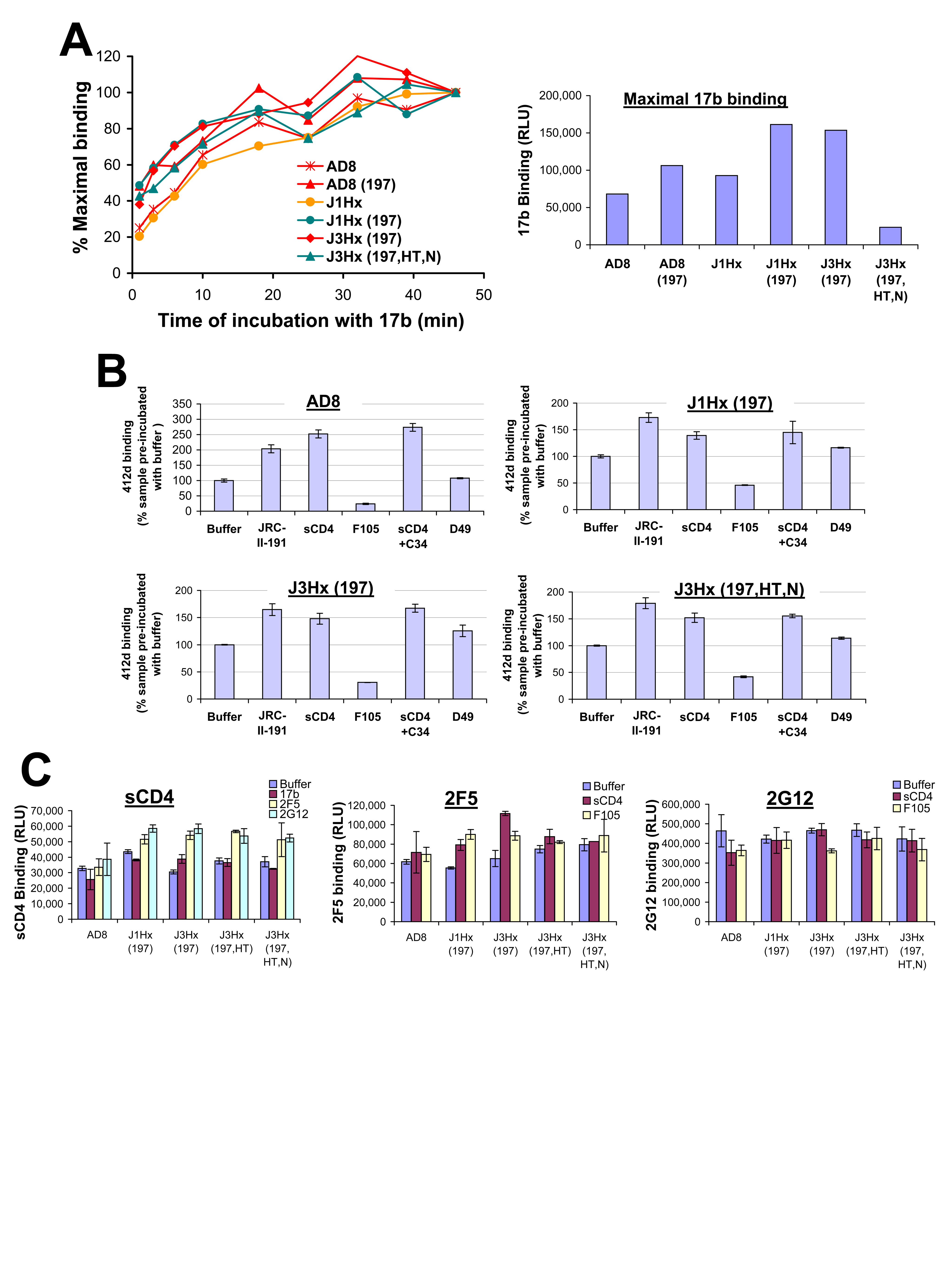

Supplement: Figure S9 — Ligand binding to cell surface-expressed envelope glycoproteins. (A) Binding kinetics of 17b antibody to envelope glycoprotein trimers expressed on the surface of cells. COS-1 cells expressing the indicated envelope glycoprotein variants were incubated with the 17b antibody (2 µg/ml) at 37°C for different time periods. Cells were then washed to remove excess antibody and binding detected using a secondary HRP-conjugated antibody. Data are presented as a percentage of maximal binding measured at the last time point. Envelope glycoproteins are color coded according to their sensitivity to neutralization by 17b. Absolute binding values measured at the last time point are shown in the panel on the right. (B) Effect of inhibitor binding to envelope glycoprotein trimers on recognition by the 412d antibody. COS-1 cells expressing the indicated envelope glycoprotein variants were incubated for 30 min at 37°C with JRC-II-191 (200 µM), sCD4 (400 nM), the F105 antibody (10 µg/ml), sCD4 and C34 peptide (400 nM and 1 µM, respectively) or the D49 antibody (10 µg/ml). Cells were then washed three times and incubated with the 412d antibody (0.7 µg/ml). 412d binding was detected using a preparation of HRP-conjugated goat-anti-human IgG secondary antibodies that does not bind to the F105 antibody or to the mouse D49 antibody. Data represent mean binding values (± SEM) derived from duplicate samples. As expected, initial incubation with sCD4 or the CD4-mimetic compound JRC-II-191 increased 412d binding, whereas pre-incubation with the F105 antibody significantly decreased 412d binding, as previously shown (references 45,84). (C) Effect of inhibitor binding to envelope glycoprotein trimers on recognition by 2F5, sCD4 and 2G12. COS-1 cells expressing the indicated envelope glycoprotein variants were incubated with the priming antibodies F105, 17b, 2F5 or 2G12 (all at 5 µg/ml) or with sCD4 (20 µg/ml) for 30 min at 37°C. Cells were then washed three times and incubated with sCD4 (left pan [file ppat.1002101.s009.tif]

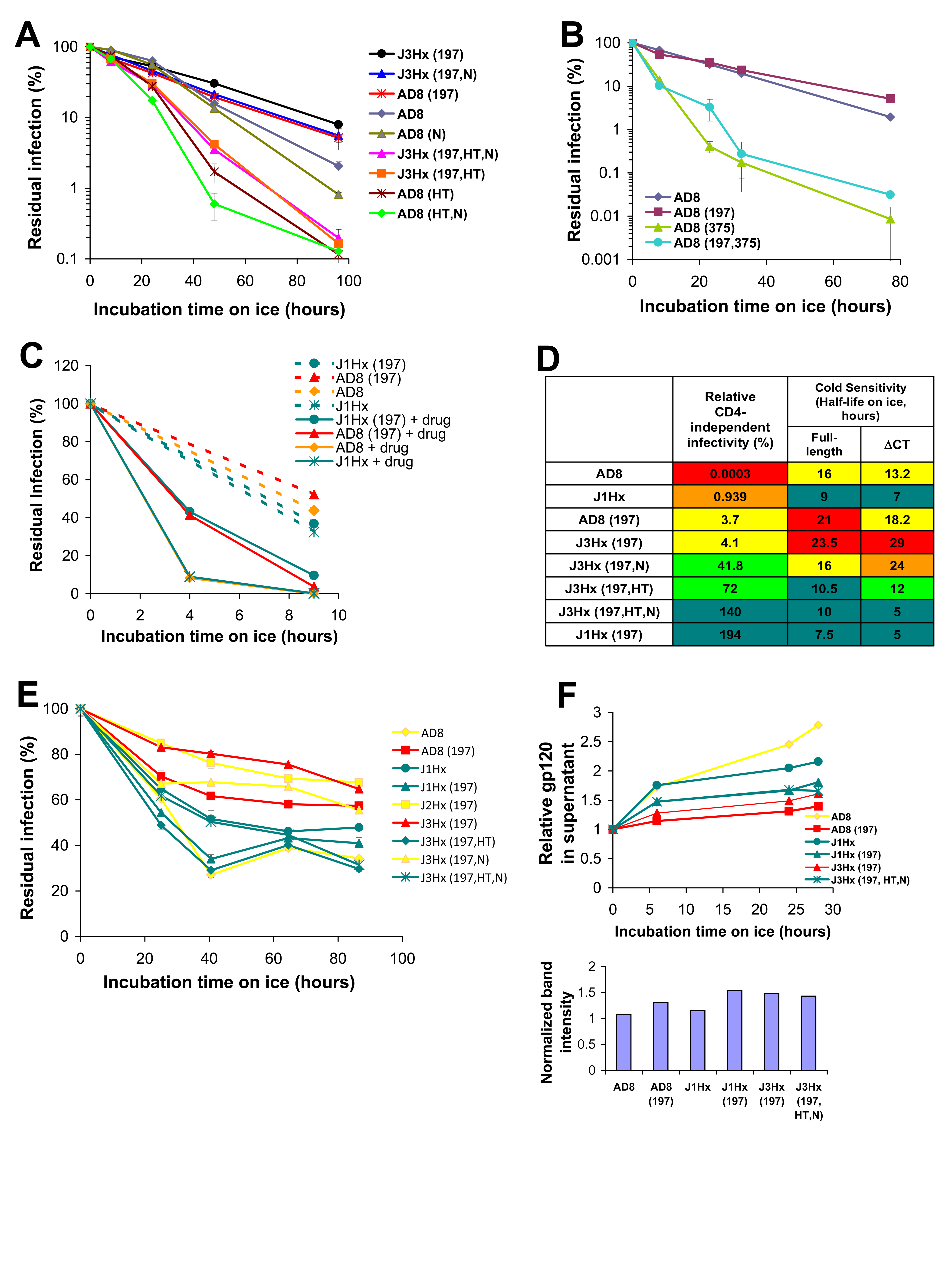

Supplement: Figure S10 — CD4 independence and cold sensitivity of envelope glycoproteins. (A) Sensitivity of the indicated envelope glycoprotein variants to cold-induced inactivation. The level of infection of CD4+CCR5+ cells by viruses with the indicated envelope glycoproteins following incubation on ice for the times shown was divided by the level of infection seen for freshly thawed viruses to yield the residual infection. Data represent mean levels of infection (± SEM) derived from three replicate samples. (B) Effect of the S375W change in gp120 on cold sensitivity of viruses containing the indicated envelope glycoproteins. Cold sensitivity was measured as in (A). Data represent mean percentages of residual infection (± SEM) derived from three replicate samples. (C) Effect of the CD4-mimetic compound JRC-II-191 on cold sensitivity. Viruses containing the indicated envelope glycoprotein variants were incubated for different time periods on ice in the absence or presence of JRC-II-191 (20 µM). Samples were then added to CD4+CCR5+ cells and infectivity was measured 2 days later. Residual infection represents the level of infection, relative to that observed for viruses not incubated on ice. (D) Comparison of the cold sensitivity of viruses containing full-length and cytoplasmic tail-deleted (Δct) envelope glycoproteins. In the left column, the relative level of CD4-independent infectivity of the full-length variants is indicated. Values are color coded according to the rank order of the measured phenotype. (E) Virus capture assay to measure the presence of gp120 on virions. Viruses that contain both the indicated full-length envelope glycoprotein variants and the vesicular stomatitis virus G protein were incubated for different time periods on ice and then captured on a plate coated with the 2G12 antibody. The amount of virus captured was assessed by measuring infectivity on CD4−CCR5− Cf2Th cells. A detailed description of the method is provided in the Materials and Methods. Data represent [file ppat.1002101.s010.tif]

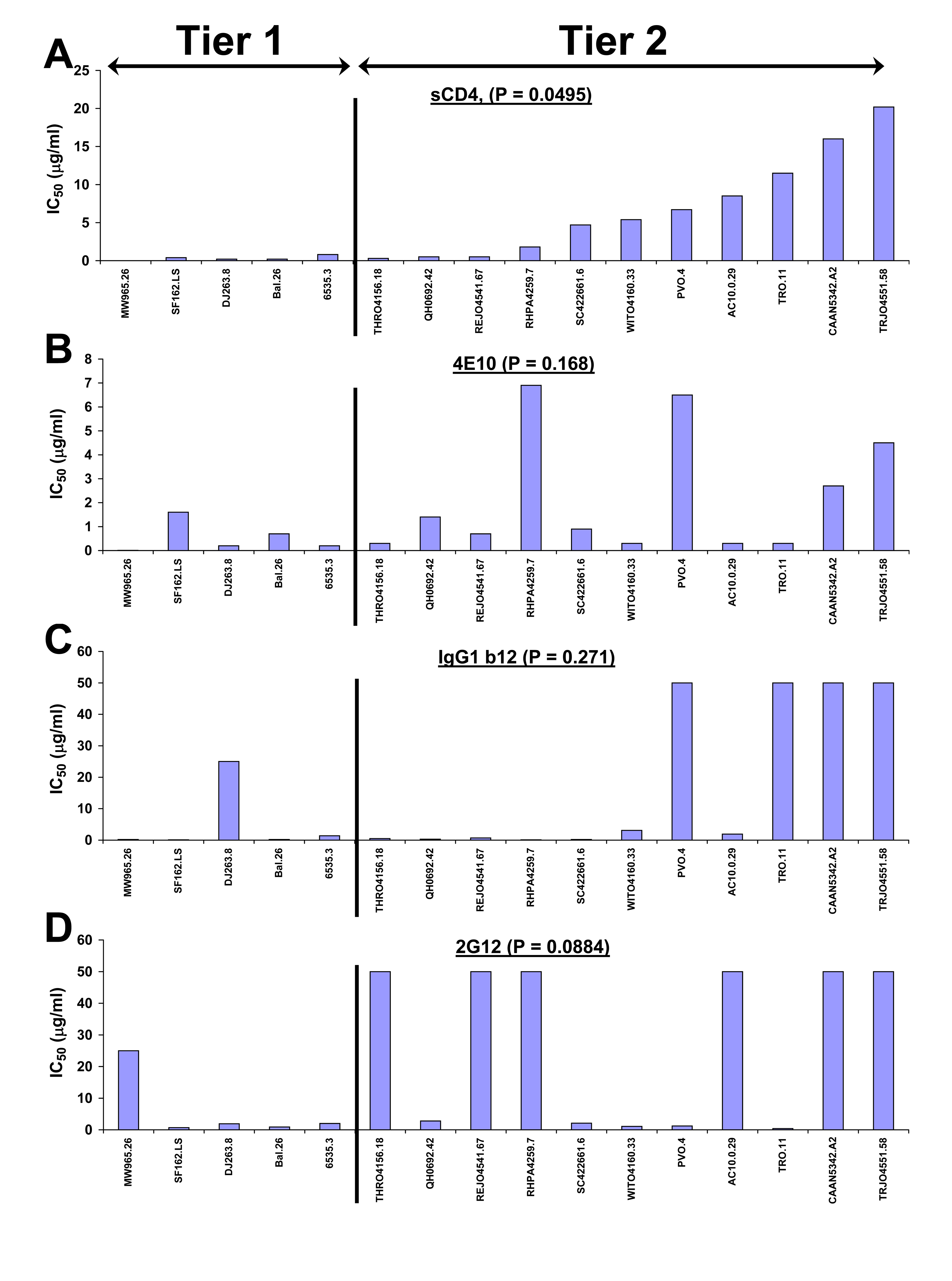

Supplement: Figure S11 — Neutralization sensitivity of a standardized panel of 16 envelope glycoproteins from primary HIV-1 isolates. Data were obtained from a registry of molecularly cloned HIV-1, SIV and SHIV envelope glycoproteins that can be used to pseudotype HIV-1 in neutralizing antibody assays; the registry was compiled by the Laboratory for AIDS Vaccine Research & Development, Duke University Medical Center and is available at http://www.hiv.lanl.gov/content/nab-reference-strains/html/home.htm). Data are presented as the concentration of inhibitor at which virus infectivity was reduced by 50% relative to cultures without added inhibitor. The two-tailed T-test P values for the comparison between the IC50 values for Tier 1 and Tier 2 envelope glycoproteins are indicated. (TIF) [file ppat.1002101.s011.tif]

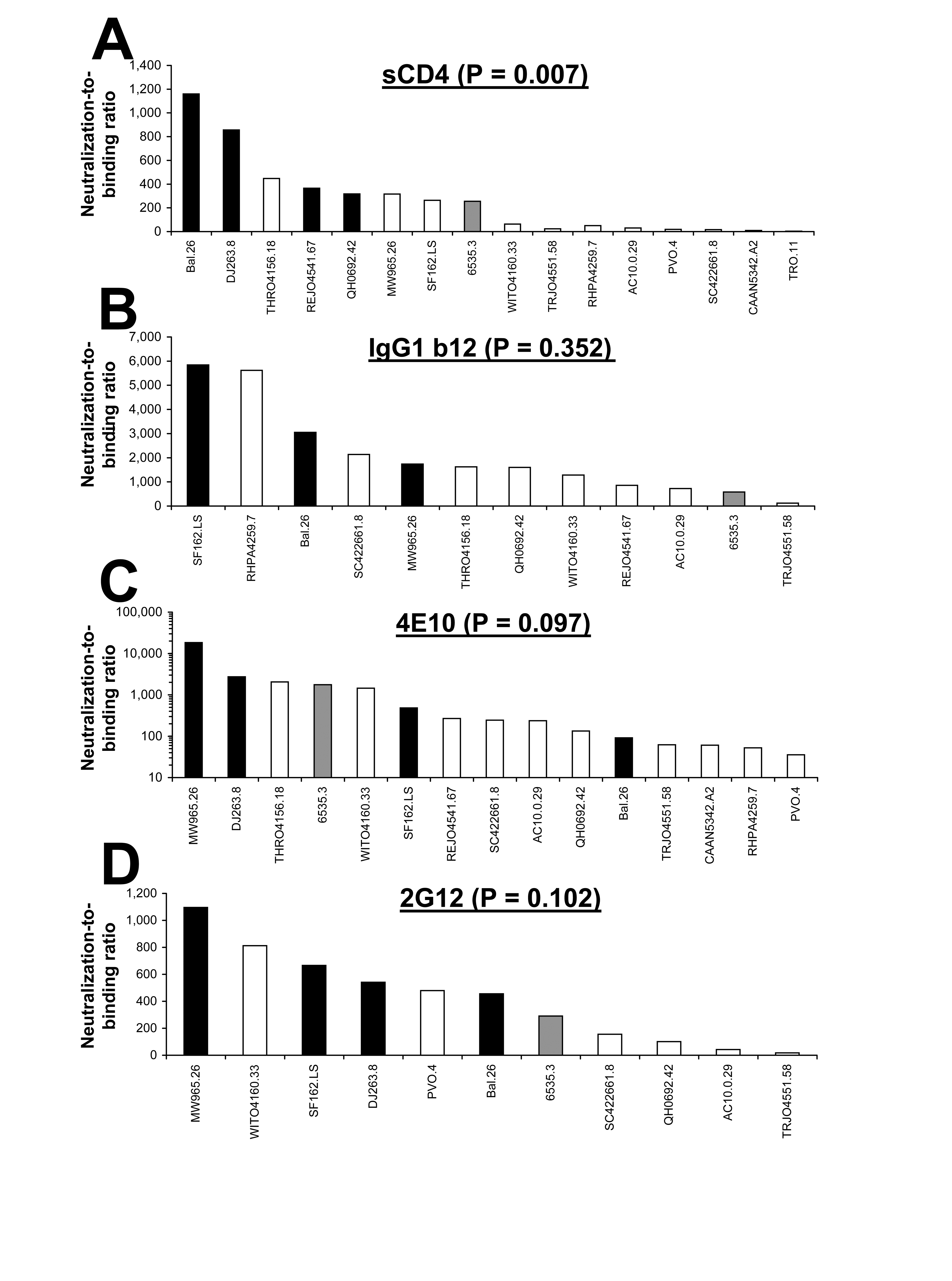

Supplement: Figure S12 — The neutralization-to-Binding Ratio as a measure of envelope glycoprotein reactivity to inhibitor binding. (A–D) Neutralization-to binding ratios for the four inhibitors. Binding of the inhibitors to cell surface-expressed envelope glycoproteins was measured by cell-based ELISA and normalized for cell-surface expression measured by cell-surface immunoprecipitation. Data are expressed as the reciprocal of the IC50 value of each envelope glycoprotein strain divided by the normalized binding efficiency. The envelope glycoproteins are colored according to neutralization sensitivity (Tier 1A, black; Tier 1B, grey; and Tier 2, empty bars). The P value for the two-tailed T-Test performed to compare the neutralization-to-binding ratios between Tier 1 and Tier 2 envelope glycoproteins is shown. (TIF) [file ppat.1002101.s012.tif]

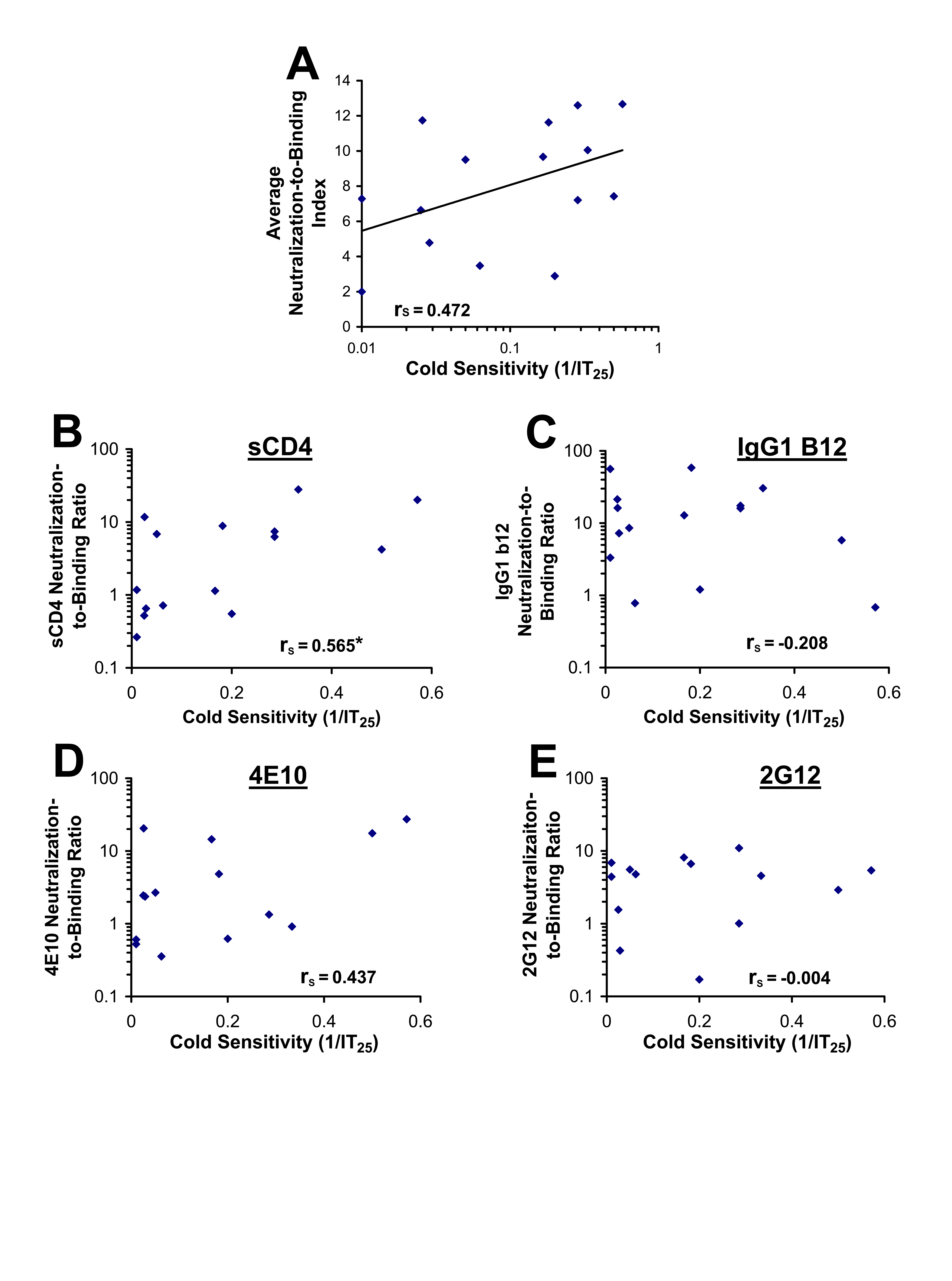

Supplement: Figure S13 — Relationship between cold sensitivity and inhibitor reactivity. (A) The relationship between the cold sensitivity of viruses containing envelope glycoproteins from the standardized panel of 16 primary HIV-1 isolates and the average neutralization-to-binding index for the four inhibitors (see Figure 8B) is shown. (B–E) The relationship between cold sensitivity and reactivity to the binding of the indicated inhibitors is shown. The neutralization-to-binding ratio is defined as the reciprocal of the IC50 value of virus containing that envelope glycoprotein divided by the normalized binding efficiency of the inhibitor to the cell surface-expressed envelope glycoproteins. The Spearman rank-order correlation coefficient is indicated (*, P<0.05). (TIF) [file ppat.1002101.s013.tif]

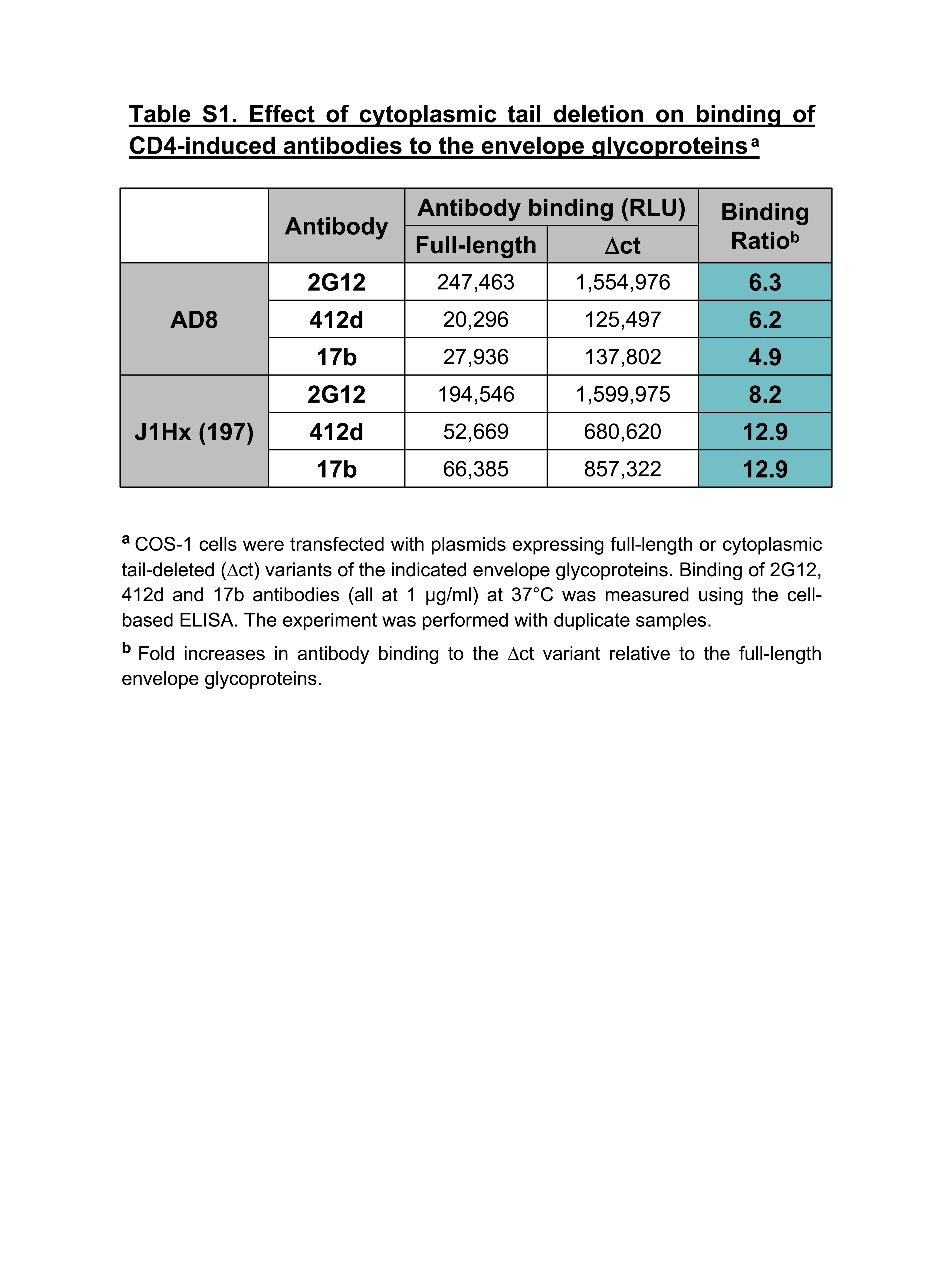

Supplement: Table S1 — Effect of cytoplasmic tail deletion on binding of CD4-induced antibodies to the envelope glycoproteins. (TIF) [file ppat.1002101.s014.tif]
